# Interorgan communication with the liver: novel mechanisms and therapeutic targets

**DOI:** 10.3389/fimmu.2023.1314123

**Published:** 2023-12-12

**Authors:** Jiulu Zhao, Xi Zhang, Yuan Li, Jizhang Yu, Zhang Chen, Yuqing Niu, Shuan Ran, Song Wang, Weicong Ye, Zilong Luo, Xiaohan Li, Yanglin Hao, Junjie Zong, Chengkun Xia, Jiahong Xia, Jie Wu

**Affiliations:** ^1^ Department of Cardiovascular Surgery, Union Hospital, Tongji Medical College, Huazhong University of Science and Technology, Wuhan, China; ^2^ Center for Translational Medicine, Union Hospital, Tongji Medical College, Huazhong University of Science and Technology, Wuhan, China; ^3^ Department of Anesthesiology, Union Hospital, Tongji Medical College, Huazhong University of Science and Technology, Wuhan, China; ^4^ Key Laboratory of Organ Transplantation, Ministry of Education, National Health Commission Key Laboratory of Organ Transplantation, Key Laboratory of Organ Transplantation, Chinese Academy of Medical Sciences, Wuhan, China

**Keywords:** liver, organ communication, hepatokine, immunity, immune homeostasis, immune cells, immune related diseases

## Abstract

The liver is a multifunctional organ that plays crucial roles in numerous physiological processes, such as production of bile and proteins for blood plasma, regulation of blood levels of amino acids, processing of hemoglobin, clearance of metabolic waste, maintenance of glucose, etc. Therefore, the liver is essential for the homeostasis of organisms. With the development of research on the liver, there is growing concern about its effect on immune cells of innate and adaptive immunity. For example, the liver regulates the proliferation, differentiation, and effector functions of immune cells through various secreted proteins (also known as “hepatokines”). As a result, the liver is identified as an important regulator of the immune system. Furthermore, many diseases resulting from immune disorders are thought to be related to the dysfunction of the liver, including systemic lupus erythematosus, multiple sclerosis, and heart failure. Thus, the liver plays a role in remote immune regulation and is intricately linked with systemic immunity. This review provides a comprehensive overview of the liver remote regulation of the body’s innate and adaptive immunity regarding to main areas: immune-related molecules secreted by the liver and the liver-resident cells. Additionally, we assessed the influence of the liver on various facets of systemic immune-related diseases, offering insights into the clinical application of target therapies for liver immune regulation, as well as future developmental trends.

## 1 Introduction

The liver is an important, multifunctional organ that serves as a central hub for numerous physiological processes. It is involved not only in the synthesizing, transforming, and decomposing of proteins, carbohydrates, lipids, and vitamins within the body, but also participates in the transformation and detoxification of hormones, drugs, and other compounds (1). Additionally, the liver has the functions of bile secretion, phagocytosis and immune defense (1, 2). Notably, the liver comprises the largest reticuloendothelial phagocytic system in the human body. The hepatic sinusoid contains a large number of Kupffer cells, which can engulf exogenous substances, pathogenic microorganisms, and other particulate matter present in the blood (3, 4). In the event of infection-related mucosal damage, pathogenic substances within the intestine can breach the intestinal mucosal barrier, the primary defense line of the intestinal immune system, and access the capillaries and lymph vessels in the intestinal wall (5, 6). Subsequently, the mesenteric lymph nodes and the liver serve as the second line of defense for the intestinal immune system (7). Under typical conditions, the liver’s unique anatomy and cellular composition bestow it with immune defense and immune regulation functions. Serving as a pivotal defense barrier between the body’s internal milieu and the external environment, the liver contributes an array of secreted proteins (including hepatokines, plasma proteins, inflammatory factors, and complements, etc.) that critically participate in the regulation of immune response (8, 9).

Within this review, we summarize the effects of liver remote immune regulation on immune cells and immune homeostasis. Beginning with the distinctive anatomical structure and cellular components of the liver, we describe the intricate orchestration of innate and adaptive immune responses through various liver secreted proteins and intrahepatic immune cells. Moreover, we synopsize the regulatory mechanisms within the liver under diverse pathological conditions, with the aspiration that this review will furnish valuable insights towards a better understanding of hepatic immune modulation.

## 2 Liver hemodynamics and histology

### 2.1 Hemodynamics of the liver

The adult liver typically weighs between 1 to 2.5 kg and has a V-shaped structure with a reddish-brown coloration. The majority of the liver resides within the right hypochondriac and epigastric regions, while a smaller portion extends into the left hypochondriac region, and is protected by the ribs and costal cartilage. The concave face of the liver interfaces with the abdominal viscera (10). The liver encompasses the Glisson system as well as the hepatic venous system; within the Glisson system are the portal vein and the proper hepatic artery. The liver also boasts an abundant blood supply, with its blood volume accounting for approximately 14% of the total human body. Blood flow in an adult liver range from 1500 to 2000 ml per minute and, unlike other abdominal organs, the liver receives a dual blood supply. The hepatic artery carries oxygenated blood from the heart, while the portal vein gathers venous blood rich in nutrients from the digestive tract (11).

The liver receives approximately 1/4 of cardiac output (12). The liver vasculature can be categorized into hepatic vessels and effluent hepatic vessels. Blood entering the liver runs through the hepatic artery and hepatic portal vein, constituting a dual vascular supply. The effluent hepatic vessels constitute the hepatic venous system. Approximately 1/4 of the liver blood supply is derived from the hepatic artery, which carries oxygenated blood and antibodies from the heart. Upon entering the liver, arterial blood divides into branches at various levels, eventually reaching the interlobular artery (13). The hepatic portal vein serves as a pivotal conduit for the liver, contributing approximately 3/4 of the liver’s blood supply. This vessel transports nutrient-laden blood from the gastrointestinal tract to the liver for metabolism (14). The blood, mixed with arterial blood, flows into sinusoidal vessels of the liver. As capillary-like structures, the flow rate of blood in these vessels is approximately half that of other capillary counterparts, which facilitates the detection of the specific molecules or pathogens by immune cells (15). The hepatic portal vein originates from the convergence of the splenic vein and the superior mesenteric vein. The portal vein additionally exhibits lateral anastomosis with the vena cava, though these channels are typically not open (16). The interconnectedness of these blood vessels means that liver-related pathological factors (e.g., cirrhosis) disrupting portal vein circulation can result in blood stasis, potentially leading to splenic congestion and hematoma formation. In instances where collateral circulation becomes open, as seen in esophageal and gastric varices, or in the event of rupture and hemorrhage, anastomoses between the portal vein and inferior vena cava via the rectal venous plexus may prompt rupture of the plexus and subsequent rectal bleeding. Alternatively, if the portal vein establishes anastomoses with both the superior and inferior vena cava through the periumbilical venous plexus, this can lead to portal hypertension and the resultant distension of periumbilical veins (17).

**Figure 1:**
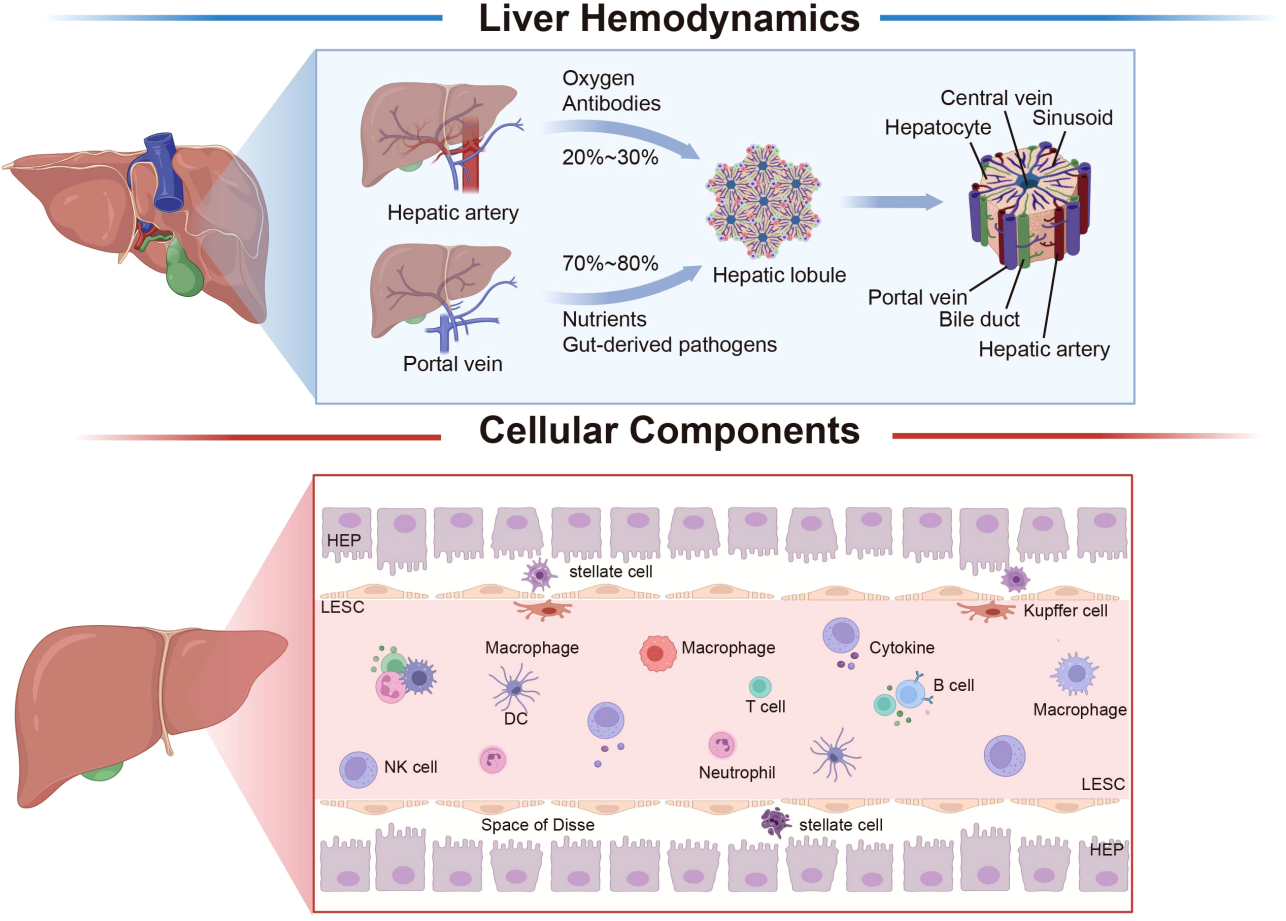
Liver hemodynamics and histology. The liver receives a dual blood supply from both the hepatic artery and the portal vein. Approximately 20–30% of the liver’s blood supply is derived from the hepatic artery, which primarily serves the purpose of oxygen delivery, while the remaining 70–80% originates from the portal vein, which is primarily responsible for nutrient supply. The fundamental structural and functional unit of the liver is the hepatic lobule, which is centered around the central vein, and hepatocytes radiate outward to form hepatic cord; between the hepatic cord is the hepatic sinusoid. The liver is composed of parenchymal cells (hepatocytes) and non-parenchymal cells, with liver non-parenchymal cells mainly encompassing LSEC, hepatic stellate cells, Kupffer cells, and immune cells. The liver’s various cell types interact, undergo precise regulation, and collaboratively perform specialized functions, collectively contributing to the biological functions of the liver. The space between hepatocytes in LSEC is the space of Disse, which is where hepatocytes and blood exchange substances. LSEC, liver sinusoidal endothelial cell; HEP, hepatocyte; DC, dendritic cell; NK, natural killer cell. Created with BioRender.com.

The hepatic artery serves as a vegetative vessel for the liver, delivering oxygen and nutrients essential for liver metabolic processes. It accounts for about 20% to 30% of the total liver blood flow is attributed to the hepatic artery, and its pressure surpasses that of the portal vein by 30-40 fold (18). The portal vein functions as a crucial conduit for the liver, constituting approximately 70%-80% of the liver’s blood supply. Characterized by lower pressure, it carries nutrient-rich blood from the digestive tract and pancreas. Upon traversing the sinusoidal space, this blood is assimilated by hepatocytes, subsequently undergoing processing. A portion is then released into the bloodstream for systemic use, while the remainder is provisionally stored within hepatocytes to serve potential demands (19) (**Figure 1**). Hepatocytes are usually divided into three regions within the hepatic lobules. Hepatocytes close to the portal vein are characterized by increased gluconeogenesis and beta oxidation, which results from toxins and microorganisms of gut origin, as well as blood rich in nutrients and oxygen. Conversely, hepatocytes close to the central venous are exposed to lower concentrations of nutrients and oxygen, which are associated with detoxification, enhanced glycolysis, and lipogenesis (20).

### 2.2 Cellular components of the liver

#### 2.2.1 Hepatocytes

Hepatocytes constitute a vital component of the liver and perform approximately 70% of its essential functions (21)(**Figure 1**). Hepatocytes also create a crucial cellular barrier, which separates sinusoidal blood from tubular bile. Hepatocytes exhibit distinct tissue polarity, positioning their basement membrane towards hepatic sinusoidal endothelial cells, while their apex generates numerous bile canaliculi in direct juxtaposition with neighboring hepatocytes (22). Another hallmark of hepatocyte morphology is the creation of subendothelial positive sinusoidal spaces (Disse spaces) along both facets of the hepatocyte basal surface (23). Hepatocytes display spatial heterogeneity, with their distribution within hepatic lobules dictating their distinct functionalities (20). While liver cells in a healthy liver exhibit slow regeneration, they can undergo rapid proliferation in response to liver damage (24). Previous studies have suggested that hepatocytes exhibit a stable phenotype (25). However, recent research highlights the phenotypic plasticity of hepatocytes. For example, hepatocytes can convert into biliary-like cells in response to cholestatic injury and liver cancer (26). Moreover, the newly discovered NOTCH-YAP1/TEAD-DNMT1 axis is crucial for hepatocyte transdifferentiation (27).

#### 2.2.2 Liver sinusoidal endothelial cells

LSECs constitute approximately 50% of the non-parenchymal liver cell population, making them the predominant non-parenchymal cell type in the liver (28). Hepatic sinusoidal endothelial cells form a lining along the low-shear sinusoidal capillary channels within the liver. As opposed to typical capillaries, LSECs feature window-like pore structures and the absence of a continuous basement membrane beneath the endothelium. This unique arrangement permits the formation of an unobstructed conduit between sinusoidal blood and the Disse space, facilitating the exchange of substrates between the bloodstream and liver parenchyma and regulating the movement of lipoproteins to and from hepatocytes (29) (**Figure 1**). LSECs stand out not only as the most permeable type of endothelial cell among mammals, but also exhibit a remarkable capacity for endocytosis. The primary receptors facilitating endocytosis include the mannose receptor, scavenger receptor, and Fcγ receptor IIb2, which enable the removal of circulating waste materials (30, 31). In a physiological context, LSECs contribute to the regulation of hepatic vascular tone, aiding in the preservation of low portal vein pressure and hepatic stellate cell quiescence. This function serves to counteract intrahepatic vasoconstriction and prevent the onset of fibrosis development (32). LSECs, as important contributors to the maintenance of liver homeostasis, previously lacked specific hallmark genes. However, a recent study identified Oit3 as marker for tracing LSECs, which is predominantly expressed in ECs of the midlobular liver. Subsequently, Oit3-CreERT2 transgenic mice were generated to investigate the complexity of LSECs in liver diseases, including sinus obstructive syndrome (SOS), providing insights into the intricate relationships between liver disease and systemic conditions (33).

#### 2.2.3 Kupffer cells

Kupffer cells, an intrinsic population of liver-resident macrophages, constitute 35% of non-parenchymal liver cells and represent 90% of the total tissue macrophage population (34). These cells arise from bone marrow hematopoietic stem cells or local hematopoietic stem cells within the liver and possess a capacity for self-renewal and play a role in the clearance of microorganisms from the portal vein, thereby contributing to the maintenance of liver homeostasis (35–37). Primarily situated within the hepatic sinus, Kupffer cells are situated near sinusoidal endothelial cells, hepatic stellate cells, and natural killer cells within this sinusoidal region. They demonstrate the ability to promptly respond to intestine-originating microorganisms and their byproducts, enabling the swift execution of their functions (38, 39). In the human liver, Kupffer cells encompass distinct subsets, including classical CD14^+^CD16^-^ macrophages, non-classical CD14^+^CD16^+^ macrophages, and CD16^+^ cells (40). Kupffer cells exhibit significant plasticity, with their phenotype and function modulated by their microenvironment, a phenomenon termed macrophage polarization (41). Classically activated and M1 macrophages, associated with pro-inflammatory responses, can be bound by lipopolysaccharides (LPS) alone or in conjunction with Th1 cytokines (e.g., IFN-γ, GM-CSF). They release pro-inflammatory cytokines such as IL-1β, IL-6, IL-12, IL-23, and TNF-α. In contrast, replace activated and M2 macrophages exhibit anti-inflammatory and immunomodulatory properties. Polarized by Th2 cytokines like IL-4 and IL-13, they then produce anti-inflammatory cytokines such as IL-10 and TGF-β (42).

#### 2.2.4 Hepatic stellate cells

HSCs, also referred to as Ito cells, reside within the Disse space and constitute approximately 5–8% of the non-parenchymal liver cell population (43). In typical conditions, HSCs remain quiescent, displaying a spindle-shaped, polygonal morphology characterized by numerous lipid droplets within the cytoplasm. These droplets are enriched with vitamin A (44). During embryonic development, HSCs originate from the mesenchymal components of the septum transversum and trace their lineage back to precursor mesothelial cells that infiltrate the liver parenchyma from the liver sac (45). Under normal circumstances, HSCs remain quiescent, but can also enter a proliferative state and subsequently differentiate into myofibroblasts upon receiving signals indicative of oxidative stress and inflammation (46). The hallmark indicator of HSC activation is the upregulation of contractile fibers α smooth muscle actin (αSMA, gene name ACTA2) (47). HSCs play a pivotal role in governing liver regeneration and are closely linked to the modulation of sinusoidal tension. They are increasingly recognized as the primary cell type influencing sinusoidal blood flow regulation, and additionally fulfill immunomodulatory function (48).

#### 2.2.5 Dendritic cells

Apart from LSECs and Kupffer cells, the liver’s antigen-presenting cell population also encompasses DCs. The liver contains fewer DCs than other organs. Hepatic DCs predominantly occupy the peripheral veins and Disse space, with a minority of cells dispersed within the parenchyma (49). The activation of DCs enriched in the liver necessitates FLT3L and GM-CSF (50, 51). Liver DCs can be classified into two primary subsets: myeloid (mDCs) and plasma celloid dendritic cells (pDC) (52). Approximately one-third of hepatic CD11c myeloid dendritic cells (mDCs) exhibit CD141 expression, whereas less than 5% of circulating mDCs display this marker (53).DCs in the liver manifest an immature phenotype characterized by diminished MHC-II expression and nearly negligible levels of co-stimulatory molecules (CD40, CD80, CD86) (54).

#### 2.2.6 Natural killer cells and Natural killer T cell

NK cells, functioning as effector lymphocytes of the innate immune system, exhibit two distinct subpopulations within the liver. One subset consists of liver-resident NK cells (LrNK), which localize to the hepatic sinusoids and are identified as liver type 1 innate lymphocytes (ILC1). The other subset originates from circulating classical NK cells (cNK cells), resembling those found in peripheral blood and the spleen (55–58). Hepatic NK cells exhibit differences from peripheral NK cells regarding surface marker expression, cytokine profiles, and cytotoxic capacities. In mice, approximately 5–10% of hepatic lymphocytes consist of NK1.1/DX5/CD3 NK cells, whereas in humans, nearly 50% of hepatic lymphocytes are composed of NK cells characterized by CD56 and CD16 expression (59, 60).

The liver is also rich in NKT cells, which constitute a substantial portion of its cellular composition. NKT cells encompass distinct lineages, primarily categorized into type I NKT cells, referred to as invariant NKT cells (iNKT). These cells express characteristic, semi-invariant T cell receptors (TCR) comprising Vα24-Jα18. In contrast, type II NKT cells exhibit a divergent TCR repertoire (61). In mice, the liver hosts a substantial population of NK1.1^+^ CD3^+^ NKT cells, constituting up to 30–40% of hepatic lymphocytes (in comparison to 0.5–2% in peripheral blood). Of this subset, around 80% express an invariant TCR configuration. In contrast, human NKT cells are predominantly associated with type II NKT cells (62) (**Figure 1**).

## 3 Immunomodulation of the liver

An increasing body of research has demonstrated the liver’s role as a lymphoid tissue that contributes to immune tolerance induction, local immune responses, and the establishment of immune memory in circulating blood antigens (63, 64). The liver bridges the intestinal portal vasculature system with the systemic circulation. Within the hepatic lobule, blood travels from the portal vein triad positioned in the lobular vicinity around the portal vein to the central vein via the polarized sinusoidal network. The distinctive configuration of the hepatic lobule, the diverse assembly of constituent cells, the specialized vascular architecture, and the blood flow characteristics characterized by high volume and low flow rate collectively form a distinctive immune microenvironment within the liver and confer it with a unique immune functionality. Hepatocytes possess the ability to secrete a multitude of proteins into the bloodstream, including hepatokines, acute phase proteins, complement, and more (65). Simultaneously, the liver harbors an abundant population of immune cells engaged in immune recognition and response. These cells encompass various categories: liver-resident cells, such as Kupffer cells, LSECs, DCs, and HSCs; circulating recruiting cells include NK cells, NKT cells, neutrophils, eosinophils, and monocytes (66).

### 3.1 Impact of liver-secreted molecules on immune regulation

#### 3.1.1 Hepatokines

In this section, we discuss the common hepatokines with immune regulation functions. Moreover, we provide the detailed information in **Table 1**.

**Table 1:** Overview of hepatokines that affect immune cells.cc.

Hepatokine	Site(s) of expression	Cell-cell signaling and interaction	Known effects	Refs
PCSK9	Hepatocytes	• Stimulation of macrophages; • Promote T cells infiltration	• Stimulate LDLR degradation; accumulation of cholesterol; enhance macrophages Toll receptor function • Bind to CD36 and MHCI; improve T cell proliferation and effectors	(67–76)
Hepcidin	Hepatocytes	• Induce macrophage activation • Recruitment, adhesion of neutrophils	• Hepcidin-ferrite transporter axis increases iron content; promote pro-inflammatory cytokines expression via TLR4/TRIF pathway • Inhibition T cells activation and B cell proliferation	(77–83)
FGF21	Liver, white adipose tissue, pancreas	Inhibits macrophage polarization	• Up-regulate PPAR-γ and inhibit NF-κB; reduce pro-inflammatory cytokines • Decrease Th17 mediated inflammation through STAT3/RORγt pathway	(84–86)
Adropin	Liver, Brain	• Hinders monocytes-endothelial cell interactions • Limit monocytes differentiation	• Control glucose and lipid homeostasis • Suppress inflammation; reduce cytokines secretion • Down-regulate the multifunctional inflammatory receptor CD36	(87–92)
ANGPTL4	Liver, Adipose tissue	Enhances macrophage activation	• Activation of macrophages; promote tissue infiltration • Increase C5a levels via PI5K/AKT signaling pathway • Modulation T cells metabolic programming	(93–98)
Fetuin A	Hepatocytes, embryonic cells	• Neutrophil and platelet degranulation • Induces macrophage migration	• direct antagonism against TGF-β and TNF-α mediated inflammation • Binding to Toll-like receptor 4 activates inflammatory signals	(99–106)
LECT2	Liver, leukocyte, skeletal muscle	• Activation of various immune cells, • Prevents recruitment of inflammatory monocytes • Enhanced adhesion of monocytes	• Interaction with CD209a activates macrophages and DC • Increase ICAM-1, TNF-α, MCP-1	(107–113)
Sep	Liver	Unknown	• regulate the sensitivity/resistance of peripheral tissues to insulin • Inhibits T cell proliferation • avoid excessive activation of macrophages	(114–118)
RBP4	Liver	Activation of antigen presenting cells	• Promotes CD4 proliferation by inducing APC activation via the JNK or My88/NF-κB pathway • Induces leukocyte recruitment and expression of adhesion-related pro-inflammatory molecules	(119–126)
DPP4	Liver	Binding and activation monocytes	• Maintain lymphocyte composition and function, T cell activation and co-stimulation • Decrease pro-inflammatory and increase anti-inflammatory • Mediated impairment of Treg function and M1 polarization	(127–129)

##### 3.1.1.1 Proprotein convertase subtilisin/kexin 9

**Figure 2:**
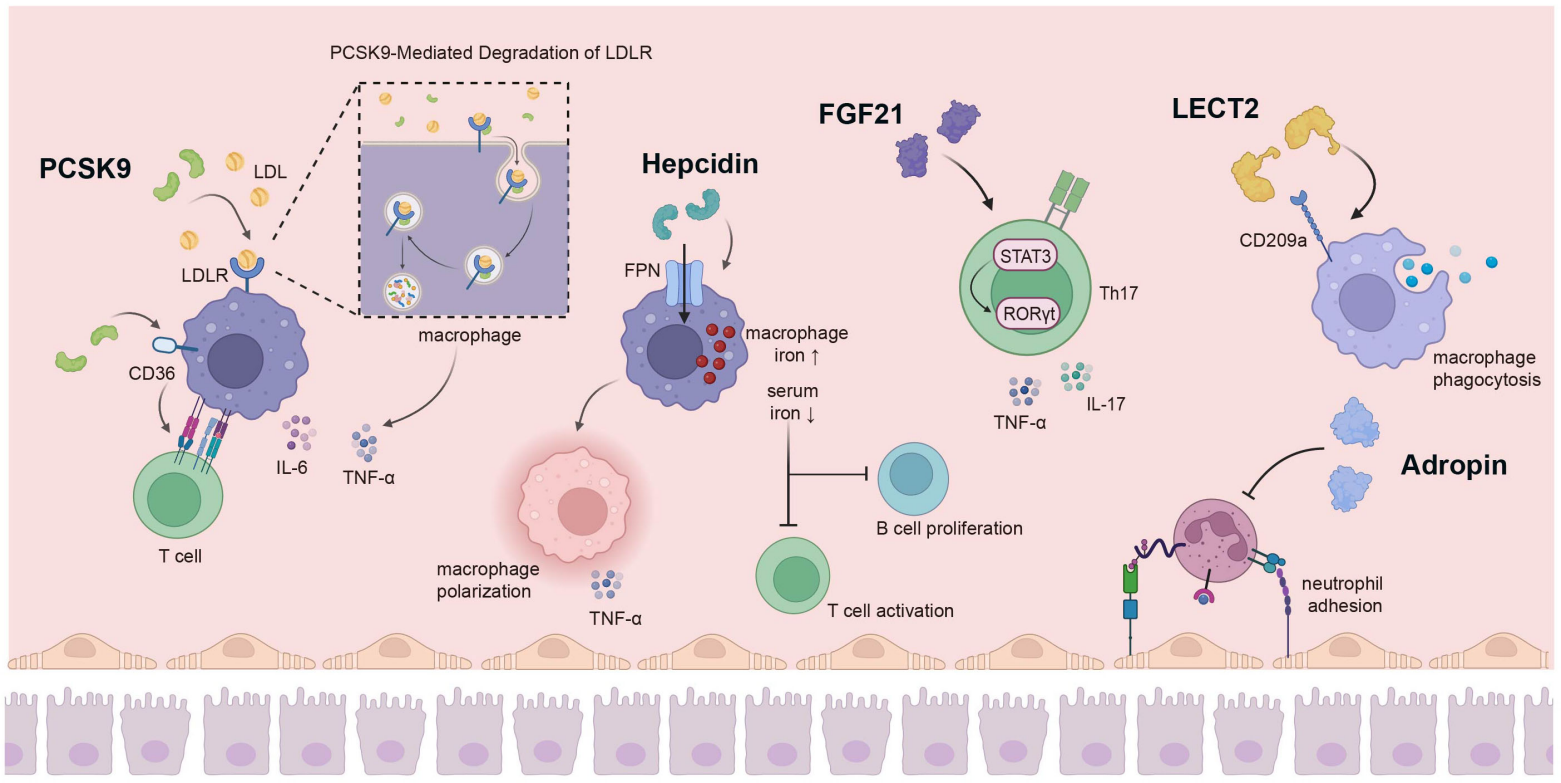
The liver secretes a variety of hepatokines for immune regulation. PCSK9 secreted by the liver has a variety of biological functions; it can promote macrophage cholesterol accumulation by stimulating LDLR degradation, affecting the production of pro-inflammatory cytokines. In addition, PCSK9 can act on CD36 on the surface of macrophages to affect the proliferation of T cells. Hepcidin can increase the iron level in macrophages by combining with FPN, and also promote the polarization of macrophages to pro-inflammatory direction, while low serum iron inhibits the activation of T cells and the proliferation of B cells. FGF21 regulates Th17-mediated inflammation through the STAT3/RORγt pathway. The interaction of LECT2 with CD209a can induce macrophage activation and enhance macrophage phagocytosis. Adropin hinders monocytes-endothelial cell interactions, thereby inhibiting the inflammatory response of endothelial cells and monocytes. PCSK9, Proprotein Convertase Subtilisin/Kexin 9; LDLR, low density lipoprotein receptor; FPN, ferroportin; FGF21, fibroblast growth factor 21; STAT3 signal transducer and activator of transcription 3; RORγt, retinoid-related orphan receptor-γt. Created with BioRender.com.

PCSK9, the ninth member of the proprotein convertase enzyme family, primarily localizes to the liver and plays a pivotal role in maintaining cholesterol homeostasis (130). Upon secretion into the plasma, PCSK9 binds to low-density lipoprotein receptors (LDLR) on the cell surface, leading to their subsequent degradation upon direct entry into lysosomes. This process actively modulates plasma LDL-C concentration (67) (**Figure 2**). Owing to its functional attributes, PCSK9 has emerged as a significant target for reducing cholesterol levels and preventing cardiovascular events (68), and an expanding body of research highlights its diverse biological functions (131). PCSK9 influences innate immunity by modulating the elimination of pathogenic LPS, a crucial component of systemic clearance and detoxification. Pharmacological inhibition of PCSK9 also has the potential to attenuate inflammatory responses and ameliorate septic shock (69, 70). Furthermore, PCSK9 can facilitate the selective buildup of cholesterol in macrophages and other immune cells. This effect is achieved through the stimulation of LDLR degradation and suppression of cholesterol reverse transport (RCT). Moreover, PCSK9 enhances lipid raft composition and bolsters Toll receptor functionality (71). Additionally, PCSK9 is capable of eliciting the synthesis of pro-inflammatory cytokines, including TNF-α and IL-6, while also facilitating the nuclear translocation of transcription factors and suppressing the generation of anti-inflammatory cytokines within macrophages (72). Aside from influencing LDLR receptors, circulating PCSK9 has the capacity to impact MHC I receptors (associated with antigen-driven immune responses) and CD36 (involved in fatty acid uptake) (132). As a target for immunotherapy, inhibiting PCSK9 can upregulate the expression of major MHC I molecules and promote the infiltration of cytotoxic T cells within tumors (73). PCSK9 hinders the recycling of MHC I to the cell surface through physical interaction with MHC I molecules, leading to their relocation and subsequent degradation within lysosomes. Inhibition of PCSK9 with small molecule compounds or monoclonal antibodies upregulates cell surface MHC I and enhances tumoral infiltrated lymphocytes (73, 74). Blocking PCSK9 improves the efficacy of anti-PD-1 therapy by promoting CD8^+^ T-cell infiltration, increasing inflammatory cytokines, and reducing Tregs (133). Additionally, PCSK9 modulates TCR recovery and signal transduction by suppressing LDLR expression, thereby impacting the immune response of CD8^+^ T cells (75). In conditions of hyperlipidemia with elevated PCSK9 levels, the increased LDLs contribute to the shift of T cells toward IL-17-producing T cells (134, 135). PCSK9 can also indirectly impact T-cell activation through oxidized LDL-induced dendritic cell maturation (136). In ankylosing spondylitis (AS), PCSK9 promotes Th1 and Th17 differentiation by activating the NF-κB pathway (137). Prior investigations by our research group demonstrated that, following heart and abdominal aorta transplantations in mice, the primary source of serum PCSK9 originated in the liver. PCSK9 influences T cell proliferation and IFN-γ production through the modulation of macrophage surface CD36 expression and the uptake of fatty acids during HTR (**Figure 2**) (76). In GVHD, the absence of PCSK9 can suppress the recruitment of macrophages and the expression of pro-inflammatory cytokines in aortic grafts. Additionally, PCSK9 knockout can hinder NLRP3 inflammasome activation, mitigate vascular smooth muscle cell (VSMC) migration and proliferation, and mitigate the development of allogeneic graft vascular lesions (138).

##### 3.1.1.2 Hepcidin

Hepcidin, alternatively referred to as the iron regulatory protein, is a cysteine-rich antimicrobial peptide synthesized in the liver. It plays a pivotal role in orchestrating iron homeostasis across the body, regulating plasma iron concentration, and governing overall iron content within an organism (139). Hepcidin-binding ferroportin (FPN) induces degradation, thereby modulating the release of iron from dietary sources, the recuperation of iron by macrophages, and the liberation of stored iron from hepatocytes into the bloodstream (77). The plasma concentration of hepcidin rises in response to iron accumulation and inflammation, while its levels are suppressed during active erythropoiesis (140). Iron thus serves as an indispensable micronutrient that is crucial for upholding cellular function. The stability of intracellular iron metabolism is intricately linked to the immune system’s functionality (141). In the context of infection and various inflammatory circumstances, hepcidin levels escalate under the influence of cytokines such as IL-6, IL-22, and others (142). Iron overload additionally reinforces host defenses, reshapes immune functionality, and modulates inflammatory response. The hepcidin-FPN axis contributes to augmented iron accumulation in macrophages and also governs pathogen proliferation within cells, representing a pivotal mechanism employed by the body to combat pathogenic infections (78, 79) (**Figure 2**). Hepcidin, derived from the liver and induced by inflammatory cytokines, reduces intestinal iron absorption and increases iron retention in macrophages. Moreover, hepcidin has the potential to prevent systemic infections of siderophilic and gram-negative bacteria by regulating iron levels (143, 144). During the early stages of infection, macrophages and neutrophils depend on hepcidin to foster the generation of pattern recognition receptor TLR4 in response to pathogens (80). The accumulation of iron within macrophages promotes their polarization towards a pro-inflammatory state; this process is coupled with the enhanced production of pro-inflammatory cytokines, including TNF-α, which is mediated by the TLR4/TRIF pathway (145). However, hepcidin-mediated hypoferremia influences leukocytogenesis, reducing the number of granulocytes but not monocytes or DCs. In addition, hypoferremia alters neutrophil effector functions and enhances mitochondrial reactive oxygen species-dependent NETosis, which is associated with chronic inflammation (146). Elevated hepcidin leads to reduced serum iron levels and can significantly compromise the immune response, influencing the reactivity of T and B cells to vaccination and infection (81) (**Figure 2**). Notably, earlier investigations have demonstrated that insufficient iron levels hinder the activation of T cells and the proliferation of B cells (82, 83). Mechanically, recent studies have proven that iron promotes the production of IFN-γ and IL-17A from CD4^+^ T cells by enhancing glucose metabolism (147, 148).

##### 3.1.1.3 Fibroblast growth factor 21

FGF21, a peptide hormone synthesized by multiple organs, governs energy equilibrium and lipid homeostasis through its interactions with FGF receptor 1 (FGFR1) and a heterodimeric complex made of β-klotho (149). The primary origin of circulating FGF21 is in the liver. It exhibits distinct metabolic functions across diverse target organs and is able to function as an autocrine, paracrine, and endocrine factor. Consequently, its biological properties are inherently complex (150, 151). FGF21 can inhibit the effect of nuclear factor NF-κB and upregulate peroxisome proliferator-activated receptor PPAR-γ on FGF receptor 1. This action mitigates the polarization of microglia and macrophages toward the M1 phenotype, consequently reducing the synthesis of pro-inflammatory cytokines (84). FGF21 induces autophagy via RACK-mediated AMPK activation and interaction with ATG5. It also further enhances cholesterol efflux and impedes the transformation of macrophages into foam cells (152). FGF21 takes on a significant anti-inflammatory role, leading to a decrease in the quantity of Th17 cells within the spleens of FGF21-treated mice. This decline is accompanied by reduced levels of IL-17, TNF-α, and IL-6, along with an increase in IL-10. FGF21 regulates Th17 mediated inflammation through the STAT3/RORγt pathway (85) (**Figure 2**). In the context of bacterial infection, the concentration of FGF21 in blood plasma rises in response to LPS stimulation. This elevation impacts innate immunity, leading to a decrease in the occurrence of endotoxemia and bacterial peritonitis (153). FGF21 also alleviates inflammation induced by LPS stimulation by impeding the TLR4/MYD88/NF-κB signaling pathway (86). In addition, FGF21 enhances the expression of Nrf2-ARE signaling-associated proteins, exerting an impact on inflammation and oxidative stress, as well as mitochondrial protection (154).

##### 3.1.1.4 Adropin

It has been suggested that Adropin, which is encoded by the energy homeostasis-associated gene (Enho), functions as a secreted protein expressed in both the liver and brain, acting as a determinant in regulating glucose and lipid homeostasis (87). Nevertheless, previous research findings indicate that Adropin may also exert a significant influence on inflammation, immune function, and neurological injuries (155). In mice lacking Adropin (Adropin knockout mice), elevated signals of F4/80, CD45, and MCP1 have been observed, along with substantial upregulation of TNFα and IL-6 genes (156). Adropin suppresses inflammation by diminishing levels of pro-inflammatory cytokines in tissues, including tumor necrosis factor alpha and interleukin-6 (88). Adropin also inhibits TNF-α induced adhesion of THP1 monocytes and endothelial cells. This inhibition of monocyte-endothelial cell interactions consequently restrains inflammatory responses in both endothelial and monocyte/macrophage compartments (89) (**Figure 2**). Furthermore, Adropin alters macrophage phenotypes towards the anti-inflammatory M2 state, as opposed to the pro-inflammatory M1 state, through the upregulation of PPAP-γ expression during differentiation from monocytes to macrophages (90). In adipose tissue, Adropin promotes the proliferation of 1T1-L2 preadipocytes by mediating ERK3/3 and AKT. Additionally, it limits the differentiation of preadipocytes into mature adipocytes, thereby curbing fat accumulation and reducing macrophage infiltration, and ultimately ameliorating inflammation (91). Moreover, deficiency in Adropin results in aberrant numbers and impaired functions of Tregs, which contributes to the development of autoimmune diseases (157). Adropin is also able to down-regulate the multifunctional inflammatory receptor CD36, which additionally provides compelling evidence for the anti-inflammatory properties of Adropin (92).

##### 3.1.1.5 Angiopoetin-like 4

ANGPTL4 belongs to the angiopoietin-like protein family and plays a pivotal role in regulating angiogenesis, lipid metabolism, glucose metabolism, and redox reactions (158). Augmented lipid uptake is linked to the stimulation of inflammation-related genes, and Angptl4 knockout mice exhibit infiltration of neutrophils and macrophages (93). Through the SIRT1/NF-κB pathway, ANGPTL4 has the capacity to modulate the expression of inflammation-related genes induced by LPS (94). ANGPTL4 can function as a downstream target of PPARβ/δ, regulating the polarization of macrophages. ANGPTL4 amplifies macrophage activation, fosters tissue infiltration, and elevates complement component 5a (C5a) levels by activating the PI5K/AKT signaling pathway, consequently leading to hypercytokinemia (C5aR, IL-6, TNF-α, and IL-1β) (95). ANGPTL4 regulates the expression of interferon-activating gene 202B (ifi202b), impacts the monocyte-to-macrophage differentiation process, and coordinates neutrophil clearance and resolution of inflammation (96). Macrophages deficient in ANGPTL4 (-/-) adopt the M1 inflammatory phenotype due to dysregulated fatty acid metabolism, leading to substantial production of TNF-α and iNOS (97). In addition, ANGPTL4 deficiency also facilitates the immunomodulation of CD8^+^ T cells through metabolic reprogramming (98).

##### 3.1.1.6 Fetuin A

Fetuin A, a heterodimeric plasma glycoprotein primarily expressed in embryonic cells and adult hepatocytes, binds to diverse receptors and demonstrates intricate physiological and pathological roles (159). Pro-inflammatory cytokines like TNF downregulate its synthesis, leading to its classification as a negative acute phase protein (99). At higher concentrations, fetal A itself exhibits anti-inflammatory effects and effectively suppresses the generation of pro-inflammatory mediators like TNF, IL-1, and nitric oxide in bacterial endotoxin-stimulated macrophage cultures (100). By interacting with fatty acids, thyroid hormones, phosphates, and calcium ions, Fetuin A engages in diverse anti-inflammatory and inflammatory functions, in addition to facilitating neutrophil and platelet degranulation and lymphocyte stimulation (101). The anti-inflammatory effect of fetal A may stem from its direct counteraction against TGF-β and tumor necrosis factor-α (TNF-α) mediated inflammation, as well as its inhibition of pathogen-associated molecular patterns (PAMP)-triggered discharge of high-mobility hinode protein 1 (HMGBP1) by innate immune cells (102). Serving as an endogenous ligand for TLR4, Fetuin A takes on a key role in innate immunity by initiating inflammatory signals (103). Fetuin A sends chemical signals that prompt macrophage migration, shifting M2 macrophages toward the M1 phenotype and inducing the secretion of cytokines (104). Fetuin A-deficient mice have previously displayed monocyte and DC cell clustering, elevated IL-12/P40, ASC1, and IL-1 β expression, and enhanced Treg upregulation (105). Functioning as an acute-phase glycoprotein, Fetuin A regulates the generation of pro-inflammatory cytokines to uphold homeostasis amidst inflammation (106).

##### 3.1.1.7 Leukocyte cell-derived chemotaxin 2

LECT2 is a hormone-like protein initially recognized as a neutrophil chemokine (160). Secreted by hepatocytes into the bloodstream, LECT2 serves as a multifunctional factor involved in numerous pathological conditions (161). In the bone marrow, LECT2 is capable of stimulating macrophages and modulating TNF expression through interaction with CD209a, consequently influencing HSC homeostasis (107). During bacteria-triggered sepsis, the interplay between LECT2 and CD209a can trigger macrophage activation, augmenting macrophage phagocytosis and bacterial eradication (108). Likewise, LECT2 pretreated DC cells can prompt cytokine secretion via the CD209a-JNK/P38 MAPK pathway (109). LECT2 is capable of hindering the recruitment and function of inflammatory monocytes, and its concentration is closely associated with inflammatory infiltration (110). In addition, LECT2 significantly enhances intercellular adhesion molecule-1 (ICAM-1) and the pro-inflammatory cytokine TNF-α, as well as monocyte chemotactic protein (MCP-1), via the CD209-JNK pathway, thereby reinforcing monocyte adherence to human endothelial cells (111). The expression of LECT2 has been shown to demonstrate a negative correlation with the immune infiltration of B cells, neutrophils, monocytes, cancer-associated fibroblasts, and myeloid DCs, while exhibiting a positive correlation with T cells, endothelial cells, and hematopoietic stem cells (112). LECT2-deficient (LECT2^-/-^) mice have exhibited a notably elevated proportion of liver NKT cells, which might contribute to the development of hepatitis (113). LECT2 also curtails the advancement of hepatocellular carcinoma (HCC) by interacting with iNKT cells, thereby obstructing β-catenin-induced inflammation (162) (**Figure 2**).

##### 3.1.1.8 Selenoprotein P

SeP, a secreted protein derived from the liver, functions to modulate the sensitivity or resistance of peripheral tissues to insulin (114). The essential trace element selenium (Se), integrated into Sep in the form of selenocysteine, contributes to diverse metabolic disorders linked to oxidative stress (163). The metabolism and regulation of selenium have significant implications for the physiological system, particularly the immune system (164, 165). In particular, deficiency in Se results in excessive generation of T cell oxidants, leading to the suppression of T cell proliferation upon stimulation of the TCR (115). Mice without SeP in T cells have been found to exhibit diminished numbers of mature and functional T cells within lymphoid tissue, as well as compromised T cell-dependent antibody responses (116). Sep also plays a crucial role in the activity of pro-inflammatory macrophages, and the suppression of SeP results in higher levels of inflammatory cytokine in tissues (117). SeP control oxidative bursts and cytokine production, enhance phagocytosis and killing, regulate inflammatory responses, and mitigate toxic damage arising from excessive macrophage activation (118).

##### 3.1.1.9 Retinol binding protein 4

RBP4, a member of the lipid carrier protein family, serves as the primary transporter of the hydrophobic molecule retinol (vitamin A) in circulation (166). RBP4 is produced in the liver and released by hepatocytes upon loading retinol and binding to the thyroxine transporter (TTR) (167). RBP4 participates in various biological activities as a vitamin A carrier, including cell proliferation, differentiation, immune regulation, bile secretion, and glucose and lipid metabolism (119, 120). Through the JNK pathway, RBP4 can activate APCs *in vivo*, leading to the proliferation of pro-inflammatory CD4^+^T cells and Th1 polarization (121). In instances of insulin resistance, RBP4 can trigger pro-inflammatory cytokine activation in macrophages via the c-Jun N-terminal protein kinase and TLR4 pathways (122). RBP4 activates innate immunity activation, leading to adaptive immunity induction. In mice with RBP4 overexpression, RBP4-induced macrophage antigen presentation and ensuing T cell activation are mediated by the MyD88 pathway, as well as downstream mitogen-activated protein kinases and NF-κB pathways (123). Furthermore, RBP4 can decrease IL-1β levels through the TLR3/MD1 receptor complex and TLR2, which activate NLRP3 inflammasomes in a glucose-dependent manner (124). Within an inflammatory microenvironment, RBP4 also enhances NOX1 and NF-κB activation, facilitates ROS accumulation, prompts M1-like polarization of Kupffer cells, and culminates in the excessive generation of inflammatory cytokines, such as TNF-α (125). Increased RBP4 levels can directly lead to endothelial inflammation, recruit leukocytes, and induce the expression of adhesion-related pro-inflammatory molecules, such as VACM-1, ICAM-1, E-selectin, and IL-6 (126).

##### 3.1.1.10 Dipeptidyl peptidase-4

DPP4 is a dipeptidyl protease that can exist as either a cell membrane protein or a soluble plasma protein and is synthesized and secreted by the liver. Concentrations of DPP4 are associated with body mass index and insulin resistance. Moreover, DPP4 can collaborate with plasma factor Xa to enhance the inflammatory responses of adipose tissue macrophages (ATM). Increased expression of DPP4 or external administration of DPP4 leads to decreased levels of pro-inflammatory IL-1β, IL-6, and IL-13, and augments the synthesis of the anti-inflammatory IL-10. In contrast, the DPP4 inhibitors sitagliptin and vildagliptin elevate the production of pro-inflammatory cytokines (127). DPP4 assumes a crucial function within the immune system, wherein it plays a vital role in upholding the composition and function of lymphocytes, as well as facilitating T cell activation and co-stimulation (128). DPP4 binds to the IGF2R receptor on the surface of Treg cells, triggering the activation of PKA/SP1 signaling. Consequently, this process impedes the degradation of IP3R2 and fosters the creation of mitochondria-associated ER membranes, leading to mitochondrial calcium overload in Tregs. As a result, DPP4 mediates the impairment of Treg functionality and the polarization of M1 microglia (129).

#### 3.1.2 Liver metabolites: bile acids, bilirubin

**Figure 3:**
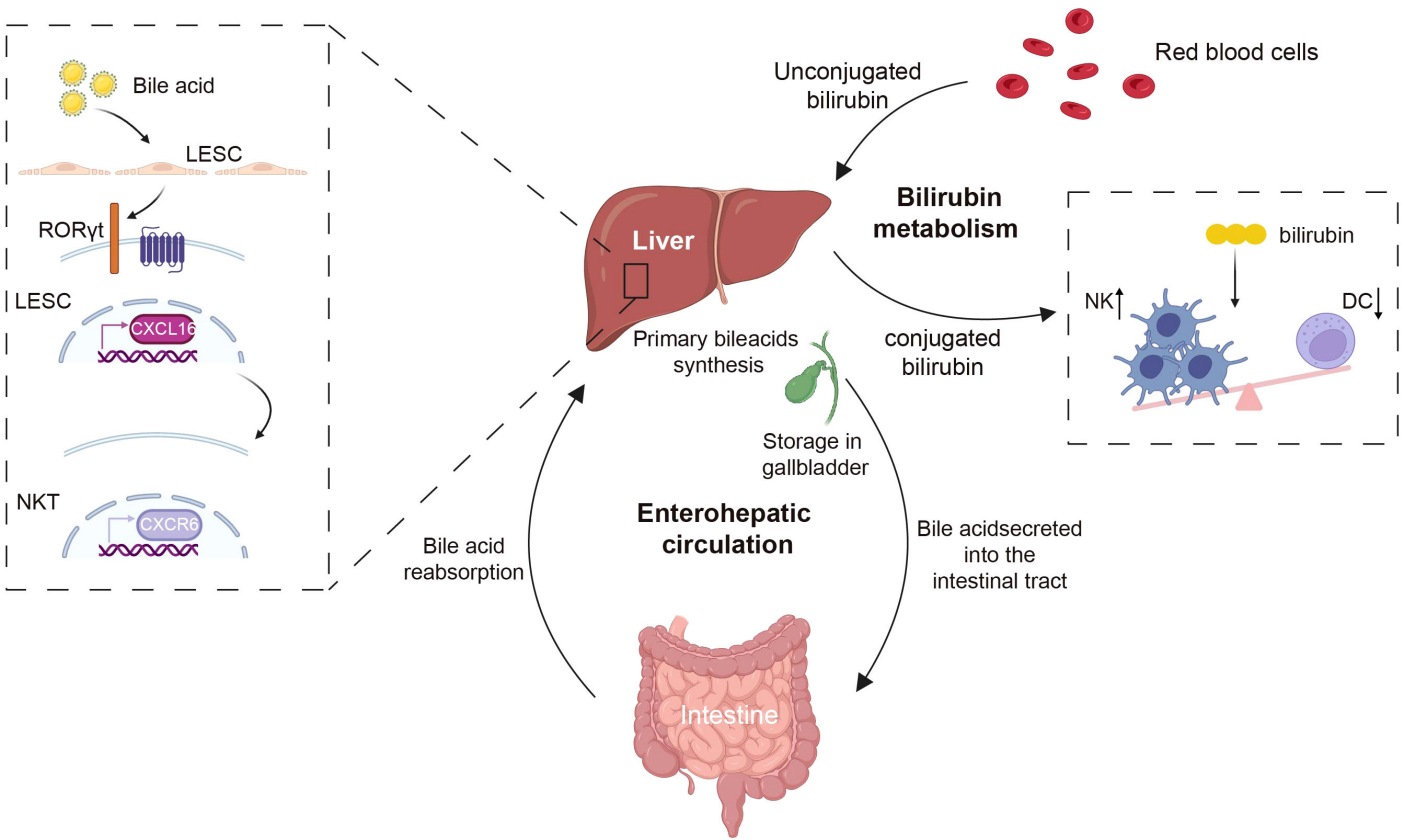
The liver metabolites of bile acids and bilirubin regulate immune cells. Bile acids, which are produced by the liver as cholesterol metabolites, are excreted into the intestine via the gallbladder, and are reabsorbed back to the liver through enterohepatic circulation. Bile acids can regulate the accumulation of CXCR6 in NKT cells by modulating the expression level of the chemokine CXCL16 on LSECs. Bilirubin, the byproduct of red blood cells, following interactions with hepatocytes, has the capacity to modulate the immune system by decreasing the population of NK cells and increasing DCs. CXCR6, CXC chemokine receptor 6; CXCL16, CXC chemokine ligand 16; NKT, natural killer T; NK, natural killer; DC, dendritic cell. Created with BioRender.com.

Bile acids are steroidal molecules formed by cholesterol oxidation in the liver, as well as signaling molecules and metabolic integrators. They activate the nuclear farnesol X receptor (FXR) and the membrane G protein-coupled receptor 5 (TGR5, also known as G protein-coupled bile acid receptor 1) in order to regulate glucose, lipid, and energy metabolism (168–170). Due to the intricate nature of bile acid signaling, its influence on the immune response is of paramount importance (171). The bile acid metabolite 3-oxocaryocholic acid (3-oxoLCA) regulates the accumulation of CXCR6 in hepatic NKT cells by suppressing the expression of the chemokine CXCL16 by RORγt receptor (172). Activation of the TGR5 bile acid receptor induces PKA kinase activity, which results in the ubiquitination of NLRP3, effectively inhibiting NLRP3 inflammasome-mediated lipopolysaccharide-induced systemic inflammation (173). Additionally, bilirubin, a product of heme metabolism in the liver, exhibits potent immunomodulatory effects, with high levels of bilirubin being able to induce apoptosis in immune cells (174). Within the context of Th17 cells, bilirubin modulates immune response by augmenting the downstream effects of AHR and by enhancing CD39 mediated *in vitro* enzymatic activity (175). Bilirubin can additionally influence cholesterol synthesis, reshaping the immune system and resulting in reduced NK cells while also promoting the expansion of DCs and MDSC populations (176) (**Figure 3**).

#### 3.1.3 Liver-derived innate immune proteins

##### 3.1.3.1 The complement system

**Figure 4:**
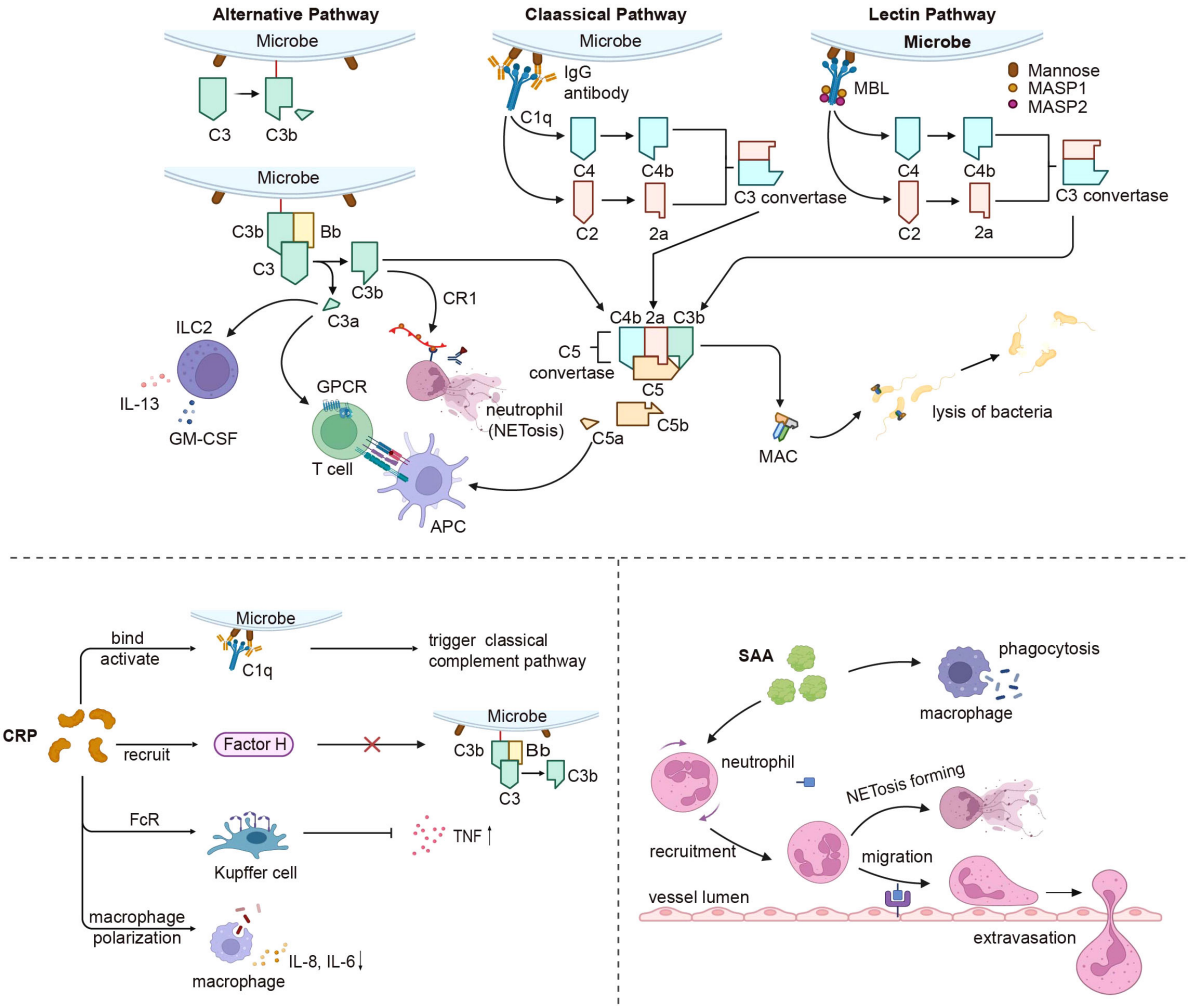
Liver derived innate immune proteins’ impact on the immune system. The complement system connects the liver and immune response after activation by the classical, alternative, and lectin pathways. All complement activation pathways eventually form MAC, which resists microbial invasion. C3a and C5a affect GPCR signaling in APC and T cells as anaphylactic toxins, while C3a also influences ILC2-mediated inflammatory responses. C3b can be recognized by CR1 to stimulate neutrophil NETosis. CRP triggers the classical complement pathway by binding and activating C1q, recruits factor H to prevent alternative pathway activation, and interacts with FcR on Kupffer cells, reducing TNF production and effects on macrophage polarization. SAA primarily increases neutrophil respiratory burst and migration, and also enhances macrophage phagocytosis. MAC, membrane attack complex; GPCR, G-protein-coupled receptors; APC, antigen presenting cell; ILC2, innate lymphoid cells; CR1, complement C3b/C4b Receptor 1; TNF, tumor necrosis factor; SAA, serum amyloid A. Created with BioRender.com.

The complement system, which acts in concert with the liver, functions as an immune complex that interconnects liver homeostasis with immune responses and various effector systems (177). Activation pathways of the complement system are categorized into three types: classical, alternative, and lectin. The culmination of all complement activation pathways is the creation of a membrane attack complex (MAC), a potent cell activator and a key driver of inflammation (178, 179). The complement system offers a range of crucial protective effects; it serves not only as the primary defense against microbial intrusion, but also contributes to diverse immune and inflammatory functions while orchestrating homeostasis (180). Additional components of the complement system apart from MA are also active. C3b can coat pathogen surfaces, enabling recognition by neutrophil complement receptor CR1 and mediating pathogen uptake, degradation, and induction of NETosis (181). Complement-derived C5a also plays a role in the recruitment of neutrophils. Upon contact with the vascular endothelium, the MAC continues to activate neutrophil extracellular traps (182). In macrophages, MAC can be internalized into endosomes, activating the inflammasome and triggering IL-1β release during inflammation (183). In addition to MAC-induced IL-1β release, C5a-C5aR1 signaling potentiates the activation of the NLRP3 inflammasome and the release of IL-1β in mouse macrophages. This was also true in human monocytes (184). Circulating C1q contributes to immune differentiation by containing monocyte specialization into dedicated antigen-presenting cells. Furthermore, it curbs the production of pro-inflammatory cytokines in innate immune cells, enhances phagocytic mechanisms in macrophages, and modulates CD8^+^ T cell mitochondrial metabolism to curtail autoantigen responses (185, 186). The complement system can also directly exert an impact on T-cell function. C3a and C3b translocate to the cell membrane and interact with costimulatory CD46, which leads to the metabolic reprogramming of T cells (187). By binding to G protein-coupled receptors on APCs and T cells, the anaphylatoxins C3a and C5a provide costimulation and survival signals to naïve CD4^+^ T cells via the PI3K-AKT pathway (188, 189). C3 and C3a have been shown to have significant roles in fostering Th2 response, while C3a additionally propels innate lymphocyte (ILC2)-mediated inflammatory reactions, eliciting IL-13 and granulocyte-macrophage colony-stimulating factors, while constraining IL-10 generation (190). Complement factor H (CFH) and complement factor I (CFI) serve as pivotal plasma regulators of the complement response. Factor H (FH) serves as a fluid-phase complement regulatory protein, preventing the hyperactivation and excessive expansion of the complement system. The negative regulator of the complement system, FH, forms complexes with nucleosomes to promote the monocyte phagocytosis-induced release of anti-inflammatory cytokines (191). Complement factor H-associated protein 1 (FHR-1), a member of the complement factor H-associated proteins (FHRs), also assumes a significant role in innate immunity by impeding complement activation through the inhibition of C5 convertase. Furthermore, FHR-1 stimulates the release of inflammatory cytokines from monocytes, exhibiting a complement-independent mechanism. The paradoxical nature of its effects confers upon it a distinct role within the innate immunity process (192) (**Figure 4**).

##### 3.1.3.2 C-reactive protein

CRP is an acute-phase protein present in minimal amounts in healthy individuals; however, it is swiftly synthesized and released into the bloodstream during bacterial infections and tissue damage (193). Previous studies suggested that CRP exists in at least two conformationally distinct forms, namely pentameric CRP (pCRP) and monomeric CRP (mCRP), which bind to distinct receptors and exhibit unique functions, respectively (194). CRP initiates the classical complement pathway by binding to and activating C1q, thereby promoting the assembly of the C3 convertase complex and mediating phagocytosis. Furthermore, CRP recruits factor H, which hinders the activation of C3b through alternative pathways (195, 196). It contains phosphocholine (PC), which efficiently mitigates ATP-induced monocyte inflammasome activation and averts inflammatory responses (196, 197). Furthermore, CRP engages with Fc receptors on phagocytes, functioning as an opsonin, to decrease TNF production while enhancing the phagocytic activity of Kupffer cells (198). CRP influences macrophage polarization; following CRP treatment, M2 macrophages transition to the M1 phenotype, accompanied by elevated secretion of IL-8, IL-6, and TNF in macrophages (199, 200). Increased CRP levels could potentially activate circulating monocytes by enhancing their chemotaxis response to MCP-1 (201, 202). CRP also exhibits anti-inflammatory effects through the upregulation of monocyte liver X receptor (LXR) α and activator receptor expression, as well as the downregulation of α2-macroglobulin expression (203). CRP induces the release of IL-8 by neutrophils via peroxynitrite-mediated activation of nuclear factor-κB and activator-1 (204). In the absence of an acute response, CRP serves a vital biological function by acting as a tonic inhibitor of the adaptive immune system, thereby maintaining peripheral T cell tolerance by inhibiting the maturation of DCs (205) (**Figure 4**).

Recent advances emphasize that pCRP exhibits both proinflammatory and anti-inflammatory properties, whereas mCRP is exclusively proinflammatory (206). The interaction of pCRP with FcγRIIa demonstrates a protective effect against autoimmune diseases by reducing the type I interferon response triggered by immune complexes (207). Both pCRP and mCRP can induce thrombus formation, activate monocytes, platelets, and neutrophils, enhance the adhesion of neutrophils and monocytes to endothelial cells, and promote the formation of neutrophil-platelet and platelet-monocyte aggregates. This process involves the production of the proinflammatory cytokines IL-1β, IL-6, and NETosis, which may result in excessive, insoluble inflammation and increased tissue damage (208). In the context of neutrophilic inflammatory responses to influenza A virus, CRP can bind to histone H4, leading to a significant inhibition of neutrophil H_2_O_2_ production, calcium influx, degranulation, and prevention of neutrophil membrane permeabilization (209). Although CRP is widely employed as a clinical marker of inflammation, it’s *in vivo* function and role in health and disease are still largely unestablished. Two recent lines of evidence form the basis for an improved model design, given the basic and conserved functional phenotypes of endogenous CRP in mice and rats. CRP gene knockout animals should be employed to investigate the *in vivo* role of human CRP. Simultaneously, the function of the human CRP is contingent upon its origin, conformation, and localization. Therefore, tissue-specific expression and conformation-locked mutants are also crucial in *in vivo* studies (210).

##### 3.1.3.3 Serum amyloid A

SAA functions as an innate immunomodulator against gram-negative bacteria, including *Escherichia coli* and *Pseudomonas aeruginosa* (211, 212). The opsonic effects of SAA mainly involve heightened neutrophil respiratory bursts, improved phagocytosis in monocyte-derived macrophages, and elevated production of TNF-α and IL-10 (213, 214). SAA performs biological functions via G protein-coupled receptor (GPCR) and formyl peptide receptor (FRP3) signaling, which leads to FRP3 activation, subsequently promoting migration of neutrophils and monocytes (215, 216). SAA fragments function alongside CCL3 to induce monocyte migration and interact with CXCL8 to facilitate neutrophil morphological alterations and chemotaxis (217). SAA induces strong endogenous stimulation of granulocyte colony-stimulating factor (G-CSF) production, which is facilitated by TLR2-mediated mechanisms (218). SAA signaling also triggers macrophages to secrete IL-1β via NLRP3 inflammasome activation, prompts dendritic cell maturation, and leads to the generation of IL-1, IL-6, PGE2, and IL-23, ultimately promoting CD4^+^ T cell secretion of IL-17A (219). As an acute-phase protein, SAA can impact insulin resistance, hepatic lipid accumulation, and liver injury by activating NF-κB signaling through its binding with TLR4 (220). Moreover, SAA participates in the crosstalk between hepatocytes and hepatic stellate cells, inducing inflammation, proliferation, and apoptosis in HSCs (221). Recent studies have discovered that SAA binds retinol and mediates the trafficking of B and T cells, as well as IgA production after bacterial infection (222, 223). In some cases, SAA may exhibit an anti-inflammatory effect (224) (**Figure 4**).

##### 3.1.3.4 Chemotokines

Apart from generating acute-phase reactive proteins, the liver also secretes chemokines that attract immune cells to sites of injury or infection in order to initiate a response (66). MCP-1 and its receptor, chemokine (C-C motif) receptor 2 (CCR2), play a critical role in recruiting and activating monocytes and macrophages at sites of tissue injury. They also modulate adhesion molecules and pro-inflammatory cytokines, such as TNFα, IL-1β, and IL-6 (225, 226). Additionally, hepatocytes secrete CXCL1 to assist in safeguarding the host against bacterial infections (227).

#### 3.1.4 Liver coagulation factors and fibrinogen

**Figure 5:**
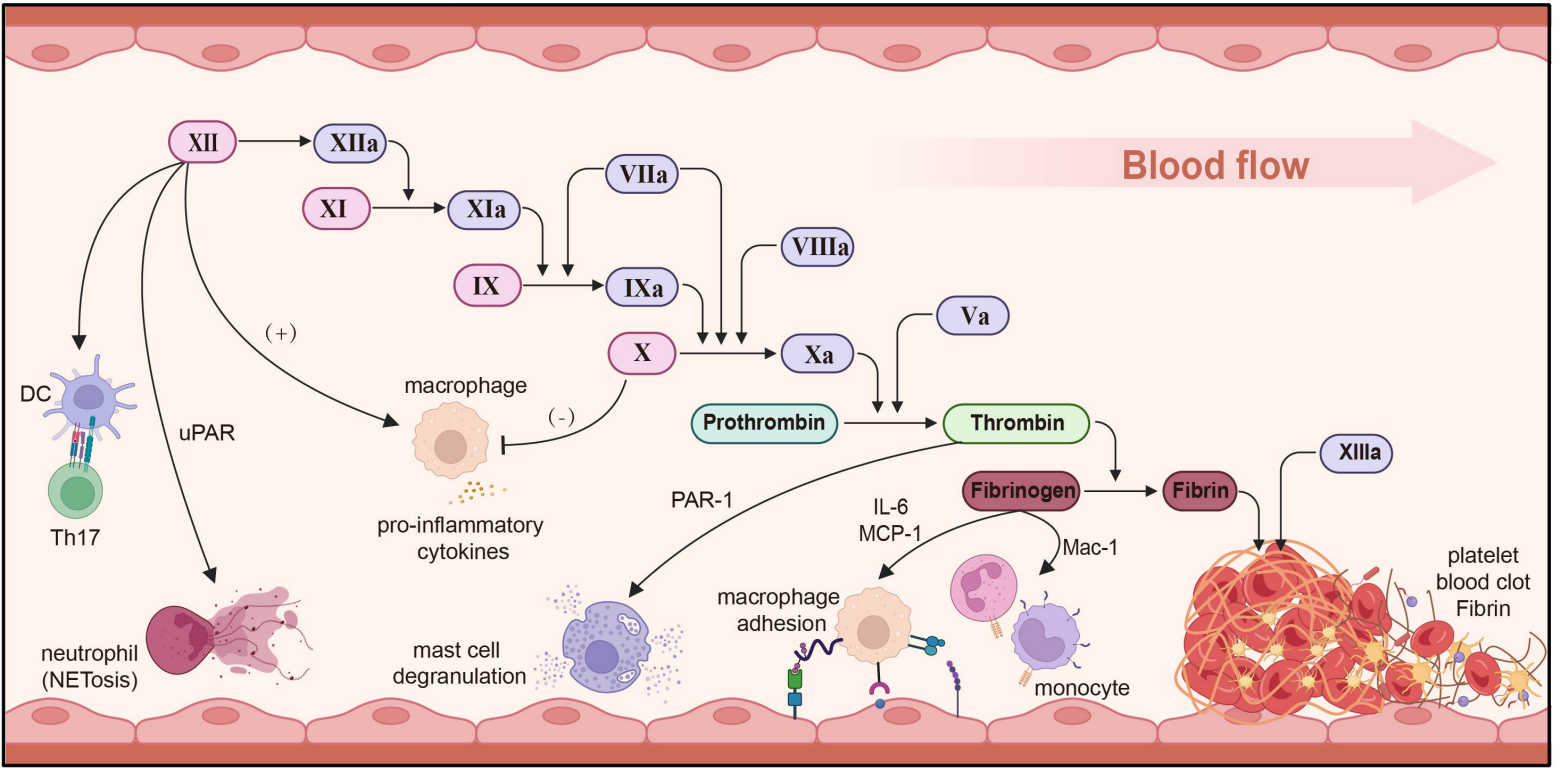
Immune regulation of the coagulation system. Factor XII stimulates DCs to induce Th17 production, promotes neutrophil adhesion and NET through uPAR, and induces macrophages to secrete pro-inflammatory cytokines. FXa inhibits and reduces macrophage secretion and reduces inflammation. Thrombin promotes mast cell degranulation via PAR-1. Fibrinogen secretes IL-6/MCP-1 and regulates macrophage adhesion. uPAR, urokinase-type plasminogen activator receptor; MCP-1, monocyte chemoattractant protein-1; DC, dendritic cell. Created with BioRender.com.

Blood coagulation, commonly known simply as coagulation, refers to the transformation of blood from a liquid state to a gel state, and is an important part of hemostasis. The coagulation process involves the sequential activation of a cascade of coagulation factors through enzymatic hydrolysis. This culminates in the production of thrombin, leading to the formation of a fibrin clot (228, 229). Recent years have seen mounting evidence indicating the involvement of coagulation factors, namely thrombin and fibrinogen, in tissue repair and inflammatory responses (230). Furthermore, the coagulation system is recognized as an integral part of innate immunity, and coagulation factors, as well as plasmin, have emerged as crucial mediators of inflammation (231) (**Figure 5**).

##### 3.1.4.1 Coagulation factors

Evidence demonstrates that interplay among coagulation factors not only contributes to hemostasis, but also significantly impacts the progression of inflammatory diseases. Factor XII, a soluble enzyme synthesized in the liver and alternatively known as the contact factor or Hageman factor, primarily serves to trigger the activation of factor XI, plasminogen, and prekallikrein. Factor XI is a key component of the intrinsic coagulation pathway, and its activation is impeded in the absence or blockade of factor II, which impairs neutrophils’ capacity to surround bacteria (232). Aside from its thrombotic function, factor FXII also fosters inflammation by activating the bradykinin release system. It can also serve as a pro-inflammatory cytokine, provoking macrophage pro-inflammatory cytokine responses. Furthermore, it triggers CD4^+^ T cell antigens to produce specific IFN-γ, and factor II prompts DCs to induce Th17 cells production via a uPAR-dependent mechanism (233–235). FXII signaling promotes neutrophil adhesion, migration, and the release of neutrophil extracellular traps through urokinase plasminogen activator receptor-mediated phosphorylation, thereby promoting NETosis (236). factor FXa can also induce the release of IL-8 and monocytes chemotaxis protein by activating protease activation of receptor 8 (237). Inhibiting factor FXa diminishes macrophage accumulation and curtails the secretion of TNF-α, COX-1, and iNOS, thereby mitigating the inflammatory response (238). Thrombin elicits vasodilation and mast cell degranulation through PAR-1 activation, while also instigating the production of cytokines/chemokines IL-6 and MCP-1, while also promoting macrophage adhesion via fibrin (ogen) (239).

##### 3.1.4.2 Fibrinogen

Fibrinogen genes are expressed almost exclusively in hepatocytes and can be stimulated during acute phase inflammation through the control of proximal promoter activity (240). In the initial stages of the innate immune response, fibrinogen bind to the bacterial surface, swiftly isolating and counteracting invading pathogens (241). Fibrinogen can invoke antibacterial effects by activating the complement system, and interacts with MBL to initiate the lectin-complement pathway (242, 243). Plasminogen and fibrinogen can also act as neutrophil surface integrin α (M) and β (2) ligands, impeding apoptosis through the activation of AKT and ERK1/2 (244). The cured fibrin matrix activates monocytes/macrophages and neutrophils through Mac-1, inciting a pro-inflammatory response (245). Fibrino-like protein 1 (FGL1) has been identified as a prominent functional ligand for LAG-3, operating independently of MHC-II. It also inhibits antigen-specific T cell activation and emerges as a potential target for the next immune checkpoint (246, 247).

### 3.2 Impact of liver cells on immune responses

**Figure 6:**
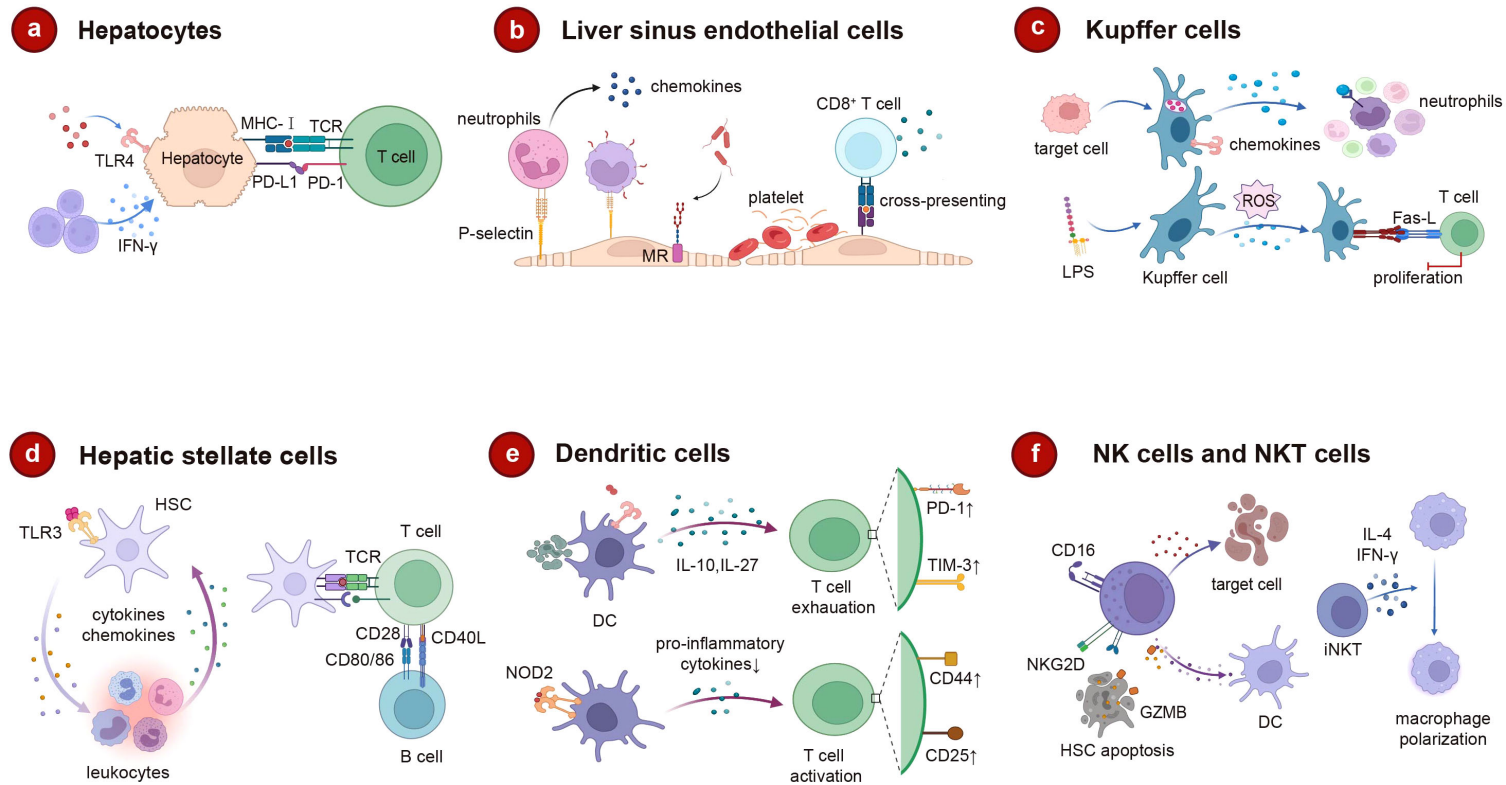
The role of intrahepatic cells in liver immune regulation. **(A)** Hepatocytes trigger adaptive immunity through MHC-I after receiving surface receptor signals. **(B)** The unique structure of LSEC promotes lymphocyte migration and platelet adhesion. As an endothelial cell with clearance effect, MR can mediate the rapid clearance of pathogens, and LSEC can cross-commission foreign antigens to CD8^+^ to induce immune tolerance. **(C)** Kupffer cells clear apoptosis target cells, promote neutrophil attachment, and also derive ROS to regulate apoptosis under the induction of LPS. **(D)** HSC not only interacts with leucocytes in a bidirectional manner, but also affects T and B cell activation as an antigen-presenting cell. **(E)** DC promotes T cell exhaustion through IL-10, IL-2. In turn, when NOD2 binds to its ligand, DC regulates T cell proliferation. **(F)** After activation, NK cells can kill target cells directly or by secreting pro-inflammatory cytokines. Increased degranulation of activated NK cells promotes HSC apoptosis through NKG2D. Activated iNKT can rapidly release IL-4 and IFN-γ mediated macrophage polarization acceleration. MHC-I, major histocompatibility complex (MHC) class I; LSEC, liver sinus endothelial cell; ROS, reactive oxygen species; LPS, lipopolysaccharide; NOD2, nucleotide-binding oligomerization domain 2; DC, dendritic cell; HSC, hepatic stellate cell; NK, natural killer; iNKT, invariant natural killer T; IFN-γ, interferon-gamma. Created with BioRender.com.

The liver harbors a significant population of immune cells engaged in immune surveillance and response. These cells fall into two main categories: liver-resident cells and circulating immune cells recruited from the bloodstream. These immune components serve a multifaceted role, aiding the liver in fending off pathogenic incursions, facilitating regeneration following injury, and supporting detoxification processes, while also instigating adaptive immune responses (248) (**Figure 6**).

#### 3.2.1 Hepatocytes

Roughly 80% of the liver’s composition is comprised of hepatocytes. While their primary responsibilities encompass material metabolism, protein synthesis, and toxin neutralization, hepatocytes also possess the capability to fulfill immune functions (249). Hepatocytes perform the synthesis of the majority of acute phase proteins and complements, serving as the foremost guardians against pathogens. Furthermore, their immune effects extend beyond this role, as hepatocytes possess the ability to adhere to the surfaces of specific microorganisms, thereby augmenting the detection of pathogens (250). Hepatocytes also possess intrinsic immune receptors that identify pathogen associated molecular pattern (PAMPs), thus instigating innate immune responses within hepatocytes. These receptors encompass cell surface receptors (e.g., TLR4), endosomal receptors (e.g., TLR3), and cytoplasmic receptors [e.g., stimulators of the IFN gene (STING)], components of the retinoic acid-inducing gene-1 (RIG-1) pathway, and members of the nucleotide-binding oligomerization domain (NOD) family) (251). Additionally, hepatocytes can also trigger an adaptive immune response. Hepatocytes possess the capacity to express MHC I molecules alongside antigen presentation-related molecules. Under inflammatory circumstances, specific hepatocytes may also be prompted to express MHC II molecules, which localize to the basolateral surface of hepatocytes. Consequently, they engage with lymphocytes and foster their activation (252, 253). Hepatic cells lack the expression of co-stimulatory molecules CD80 and CD86, making them incapable of engendering enduring activation and viability of T cells (254). Hepatocytes can also express PD-L1 in the presence of type I and type II IFN, and PD-L1 expression on hepatocytes induces apoptosis of T cells (**Figure 6A**).

#### 3.2.2 Liver sinusoidal endothelial cells

LSECs are a type of endothelial cell with a scavenging role and are responsible for eliminating soluble macromolecular waste from tissues through high-affinity endocytic receptors, such as mannose receptors (MR) and clearance receptors (SR) (255). Additionally, endocytic receptors on LSECs play a crucial role in viral infections, facilitating the swift elimination of bloodborne viruses (256). The presentation of exogenous antigens internalized by LSECs can directly influence the modulation of adaptive immune responses (257). Functioning as the principal antigen-presenting cells within the liver, LSECs promote hepatic immune tolerance and executes anti-inflammatory functions (258, 259). LSECs display an extensive array of pattern recognition receptors (PRRs), including TLR-3, 4, 7, 9, while concurrently expressing MHC molecules and co-stimulatory molecules (260). LSECs also have the capacity to present soluble antigens to naïve CD4^+^ T cells, thereby inducing regulatory phenotypes. In cirrhotic patients and mouse models of liver fibrosis, LESCs are stimulated by liver damage, which promotes the immunoproteasome LMP7 levels in LESCs and the capacity of MHCII antigen presentation to CD4^+^ T cells (261). Additionally, LSECs are capable of cross-presenting soluble foreign antigens via MHC I molecules to CD8^+^ T cells, leading to the induction of immune tolerance (262, 263). The LSEC-induced T-cell activation is influenced by the antigen load and local inflammatory factors. High antigen concentrations can overcome PD-1-mediated tolerogenic responses, leading to T-cell differentiation into effector T cells (258). Circulating CD4^+^ cells engage in repeated interactions with hepatic sinusoidal endothelial cells, thereby suppressing the secretion of inflammatory cytokines by Th1 cells and Th17 cells (264). The distinctive structure of LSECs further facilitates the migration of lymphocytes and ensures their optimal localization (265). Compared to other types of endothelial cells, the adhesion molecular expression profile of LSECs is unique, including ICAM-1, VCAM-1, VAP-1, and stabilin-1, which is crucial for leukocyte recruitment (266). Lymphocyte recruitment involves an adhesion cascade occurring within the hepatic sinuses, influenced by a low-shear environment and intercellular communication between parenchymal and non-parenchymal cells (267, 268). Integrins located on the surface of LSECs adhere to platelets and release chemokines that facilitate the recruitment of neutrophils and lymphocytes (269). In a pro-inflammatory phenotype, dysfunctional LSECs fail to maintain Kupffer cell homeostasis, leading to the release of inflammatory mediators (270). With the stimulation of mechanical stretch, LSECs release CXCL1 to promote the recruitment of sinusoidal neutrophils and to facilitate the formation of NETosis and microthrombi (271). Injured LSECs or cancer-activated LSECs (cLSECs) enhance the proliferation of Treg cells through TGF-β, which can inhibit effector T-cell function and lead to cancer progression (272) (**Figure 6B**).

#### 3.2.3 Kupfer cells

Kupffer cells, resident macrophages within the liver, play an important role in both antigen presentation and the response to tissue damage (273). Kupffer cells are central to the clearance of circulating antigens, and the presence of the complement receptor CRIg on their surface serves as a crucial element of the innate immune system. The absence of CRIg impairs the efficient clearance of circulating pathogen (274). Kupffer cells generate adhesion molecules that induce interactions with neutrophils, leading to the adherence of neutrophils to Kupffer cells and thereby assisting in the clearance of pathogens (275). Furthermore, Kupffer cells also possess the ability to eliminate activated and apoptotic cells from the bloodstream (276). LPS stimulates the production of reactive ROS by Kupffer cells, initiating the Fas-L transcription program that regulates apoptosis in T cells (277). Both Kupffer cells and LSECs contribute to the recruitment of neutrophils through the activation of TLR and CD44/HA mechanisms (278). Additionally, antigen presentation by Kupffer cells has the capacity to induce immune tolerance, leading to CD4^+^ T cell arrest and promoting the secretion of IL-10, which in turn triggers the expansion and activation of Tregs (279, 280). The chemokines generated by Kupffer cells are responsible for chemically attracting monocytes, T cells, NK cells, and DCs, thereby promoting the adhesion of T cells to endothelial cells (281). Kupffer cells have the capability to activate iNKT cells via the CD1 pathway, and this interaction is prevalent throughout the liver (282) (**Figure 6C**).

#### 3.2.4 Hepatic stellate cells

Quiescent HSCs express TLR3, which triggers the transcription and secretion of functional interferons as well as numerous other cytokines and chemokines. Upon activation into myofibroblasts, HSCs rapidly lose their ability to produce IFNγ (283). Stellate cells engage in bidirectional interactions with immune cells, whereby they not only respond to leukocyte regulation but also influence leukocyte chemotaxis and adhesion, thereby contributing to the modulation of leukocyte activation (284). As antigen-presenting cells, HSCs express MHC I and MHC II molecules, along with lipid-presenting CD1B and CD1C molecules, CD86, CD40, and other co-stimulatory molecules (285, 286). When exposed to pro-inflammatory cytokines like IFNγ, HSCs experience a notable upregulation of CD80, while the activation of CD40 results in elevated IL-8 secretion in HSCs and an increased release of monocyte chemotactic protein-1 (287, 288). Activated HSCs exhibit the expression of the negative costimulatory factor PD-L1 and engage with T cells through B7-H1-mediated apoptosis (289). HSCs may also counteract B cell activity using a similar mechanism (290). Increased CD54 expression on HSCs further contributes to the attenuation of T cell activation (291) (**Figure 6D**).

#### 3.2.5 Dendritic cells

DCs are highly immunogenic APCs that excel in capturing, processing, and presenting antigens to T cells (292). However, hepatic DCs exhibit greater tolerance compared to conventional DCs. They tend to produce IL-10 and IL-27 upon LPS stimulation, resulting in a subdued T cell response mediated by IL-27 (293, 294). IL-10 secretion by DCs can modulate the balance between Th1 and Th2 cells, augment the population of IL-4 producing Th2 cells, and enhance the generation of CD4^+^CD25^+^Foxp3^+^ Tregs (295). Furthermore, liver-resident DCs can directly lead to T cell depletion, which encompasses T cell anergy (296). DCs are classified into classical type 1 DC (cDC1), cDC2, and plasmacytoid DC (pDC) cells, of which cDC2 cells predominate in the liver and display a tolerant nature, whereas cDC1 cells engage in antigen presentation toward T cells (297, 298). The CD103^+^cDC1 subtype of DCs acts as a protective variant that impacts pro-inflammatory and anti-inflammatory balance and reduces local inflammation (299). Upon binding of NOD2 on the DC surface with its ligand, it interferes with the signaling pathways of TLR4 and TLR9 in pDC. This disruption leads to reduced secretion of pro-inflammatory cytokines, such as IL-2, IL-6, TNF-α, and IFN-γ, while concurrently inducing an upregulation of B7-H1. This modulation of pDCs activity alters the regulatory influence on T cell proliferation (300) (**Figure 6E**).

#### 3.2.6 Natural killer cells and natural killer T cell

NK cells and NKT cells are significant cellular elements within the liver microenvironment of the innate immune system. They orchestrate both cytotoxic and cytokine-mediated responses, thereby exerting a pivotal influence on the configuration of adaptive immunity (301, 302). Activation of NK cells hinges on the perturbation of the equilibrium between surface inhibitory receptors and stimulatory receptors, such as NKG2D/NKG2A (303). The NKT cell presence is essential for the activation of NK cells, contributing to the generation of IFN-γ and IL-4, which aid in facilitating NK cell activation (304). NK cells can execute direct killing of infected target cells or elicit the secretion of pro-inflammatory cytokine IFN-γ upon activation, thereby exerting cytotoxic effects (305, 306). NK cells with adequate IFN-γ levels can positively modulate CD8^+^ T cells through IFN-γ secretion, while, as the principal generators of IL-10, NK cells also contribute to the regulation of T cell activation (306, 307). The proliferation of Tregs can be additionally facilitated by NK cells through the generation of inhibitory factors like TGF-β and IL-2, which dampen dendritic cell activation. Moreover, the activated degranulation of NK cells augments and fosters hepatic stellate cell (HSC) apoptosis through a mechanism that is reliant on TRAIL and NKG2D (308). Unlike the killing mechanism of NK cells involving TRAIL and granzyme B, NKT cells predominantly operate through the release of pro-inflammatory cytokines and FasL (309). Activated iNKT cells can rapidly release IL-4 and expedite macrophage polarization through IFN-γ mediation (310). Furthermore, iNKT cells foster neutrophil infiltration via the IL-4/STAT6 pathway, whereas IFN-γ/STAT1 accelerates neutrophil apoptosis (311) (**Figure 6F**).

## 4 Liver and diseases

**Figure 7:**
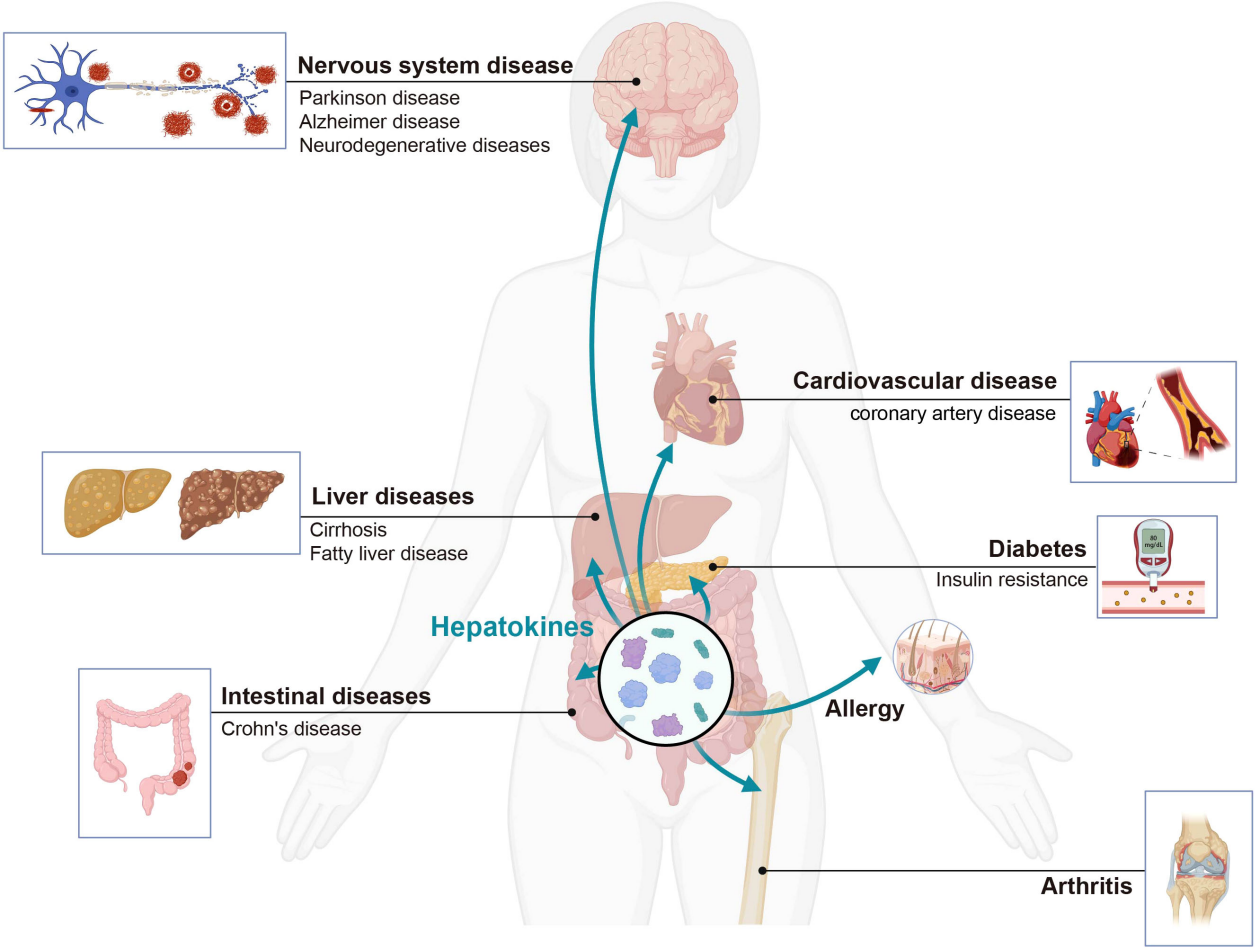
The liver secretes hepatokines effectively to regulate distant organs under different physiological and pathological conditions. Hepatokines affect the immune homeostasis of organs, and can lead to the development of various systemic diseases. Created with BioRender.com.

Advancements in liver immunology research have yielded a more profound comprehension of hepatically secreted proteins and the involvement of both parenchymal and non-parenchymal liver cells in immune responses. This progress not only contributes to unraveling the etiology of hepatitis, cirrhosis, and other hepatic disorders, but also offers novel insights into diverse, systemic immunity-linked conditions (**Figure 7**). Furthermore, it establishes a solid groundwork for the exploration and formulation of innovative therapeutics and strategies targeting liver-associated immune diseases. We summarize the detail information in **Table 2**.

**Table 2:** The impact of liver remote immune regulation on various system diseases and effective molecules.

Organ/System	Disease	Effector	Immune effect	Refs
Liver	Hepatic virus infection	APC (KC, LSEC, hepatocyte)	Antigen-presenting cells promote T cell activation and form tertiary immune structures	(312)
	Bacterial infection	KC、NKT	Blocks the activation of CD4^+^ T cells and suppresses the immune response	(313)
	Hepatitis	FGF21	Inhibits Kupffer cell activation, Prevents liver fibrosis	(314, 315)
	Hepatocellular carcinoma	Various immune cells	Hepatic tolerance and immunosuppressive properties protect tumor growth	(316, 317)
	Alcoholic liver disease	Neutrophils and NK cells	Secretion of pro-inflammatory cytokines and chemokines; induce bacterial displacement	(318)
Nervous system	TBI	Fetuin A	Inhibit microglial necrotizing apoptosis, reduce microglial activation	(319)
	Acute stroke	Bile acids	Suppress microglial activation; reduces MCP-1 and IFN-γ production	(320)
	MS	Bile acids	Prevents neurotoxicity and pro-inflammatory polarization of immune cells	(321, 322)
	Neuroinflammation	PCSK9	Inhibit microglia and astrocytes proliferation and hypertrophy	(323–325)
		Coagulation system	Activation of microglia; enhance phagocytic ability; induce peripheral macrophage recruitment	(326, 327)
	Degenerative diseases	The complement system	Remove cellular debris and apoptotic cells; promote tissue repair	(328)
Cardiovascular system	Atherosclerosis	ANGPTL4	Reduce lipid accumulation in macrophages	(329, 330)
		Hepcidin	Downregulate FPN affects intramacrophage iron	(331)
		PCSK9	Activate the TLR4/MyD88/NF-κB signaling pathway, increases pro-inflammatory cytokines	(332)
	Heart failure	Hepcidin	Decrease neutrophil activity, T cell proliferation, and impaired IL-2 production	(333)
		FGF21	Affect cardiac repair via IL-6/STAT3/MR/FGF21 axis	(334)
	Heart transplant rejection	PCSK9	Regulate CD36 expression and fatty acid uptake on macrophage surface, affect T cell proliferation and IFN-γ production	(76, 138)
Immune system	RA	FGF21	Inhibit humoral and cellular immune responses, downregulate pro-inflammatory cytokines	(335, 336)
	AS	RBP4	Inversely correlated with CRP	(337)
	Psoriasis	Sep	Anti-inflammatory	(338)
	SLE, vasculitis, Sjögren’s syndrome,	The complement system	Induce of neutrophils, monocytes, and eosinophils	(339)
Gastrointestinal tract	IBD	ANGPTL4	Affect the activation of macrophages and pro-inflammatory cytokines	(340)
		Sep	Increase intestinal inflammation	(341)
Blood system	Sepsis	Hepcidin	Defend against pathogen invasion	(342)
	PNH	The complement system	MAC destroys red blood cells, causing intravascular hemolysis	(343)

### 4.1 Liver diseases

The liver serves as an immune-tolerant organ and is proficient in upholding immune tolerance toward both self-propagated and foreign antigens entering via the portal vein. Simultaneously, it is primed to mount immune responses against pathogens and is capable of accommodating liver allogeneic grafts (9). Liver-resident macrophages are equipped to engulf particles (>200 nm) through phagocytosis, while LSECs facilitate the clearance of macromolecules (<200 nm) and colloids via receptor-mediated endocytosis (344). Additionally, KCs and LSECs present accumulated antigens to the lymphocytes, instigating T cell immune tolerance through the expression of diminished levels of MHC II and co-stimulatory molecules (345). During acute liver injury, KCs generate pro-inflammatory cytokines, such as IL-1, IL-6, TNF-α, and chemokines (346). Upon viral infection of the liver, numerous antigen-presenting cells, including KCs, LSECs, and hepatocytes, swiftly become activated. This activation initiates the triggering of specific T cell activation and expansion, ultimately culminating in the development of tertiary immune structures within the liver. In the absence of pro-inflammatory cues, these tertiary immune structures cease their regulatory function over T cells, resulting in the breakdown of CTL response. Nevertheless, under suitable signal strength, these structures can lead to persistent viral infections (312). Conversely, cytokines released by KCs in response to bacterial infection can inhibit the activation of CD4^+^ T cells, culminating in the suppression of the immune response (313). In the early phase of microbial-induced liver inflammation, type I NKT exhibit pro-inflammatory behavior, while type II NKT cells exert inhibitory effects on NKT-mediated liver injury (347). FGF21 suppresses Kupffer cell activation, reduces monocyte infiltration, and diminishes lipid-associated macrophages amidst liver inflammation, thus exerting a preventative effect against liver fibrosis (314, 315). Additionally, the liver serves as a frequent site for malignancy within the body, with HCC displaying the highest incidence among primary tumors. Typically arising as a consequence of chronic liver inflammation, these inflammatory processes synergistically instigate tumorigenesis. Upon the initiation of tumorigenesis, the liver’s tolerance and immunosuppressive mechanisms offer a protective environment for tumor growth and epithelial-mesenchymal transition. Simultaneously, the tumor microenvironment exerts additional immune suppression, ultimately driving irreversible alterations (316, 317). Numerous liver disease patterns do not stem from trauma but rather result from unhealthy lifestyles, including conditions such as alcoholic liver disease (ALD), fatty liver, and drug-induced liver injury. The release of pro-inflammatory cytokines and chemokines by neutrophils and NK cells incites the adaptive immune response, contributing to disease development. Conversely, M2 macrophages and Tregs seem to play a role in safeguarding the liver against harm (348, 349). During alcohol-induced liver damage, alcohol consumption can potentially heighten intestinal permeability, subsequently resulting in bacterial translocation and escalated LPS levels. Beyond their direct impact on liver cells and immune cells, TLRs also possess the ability to stimulate innate immunity, thereby instigating hepatic lipid droplet deterioration and ultimately contributing to liver fibrosis (318).

Furthermore, recent studies have reported that innate immune proteins derived from the liver also contribute to liver-related diseases. In acetaminophen-induced liver injury (AILI), CRP is considered a crucial checkpoint that protects against acute liver injury by preventing excessive complement activation (350). Clinical studies on acute liver injury in COVID-19 have revealed a correlation between CRP and liver damage (351). The role of complement in nonalcoholic fatty liver disease (NFLD) and ALD is complex. For instance, C3, factor B, and factor D activate the alternative pathway, leading to the generation of anaphylatoxins C3a and C5a, which induce insulin resistance and disrupt lipid metabolism in the liver (352). C1q contributes to liver injury by activating the classical complement, whereas factor D protects against ethanol-induced inflammation and promotes hepatic healing and recovery (353, 354). Several experimental models have demonstrated that complement inhibition is beneficial for liver injury (including IRI), liver transplantation, and acute liver failure. For example, anti-C5 therapy after liver transplantation can inhibit antibody-mediated rejection, improve long-term animal survival, and reduce biliary injury and liver fibrosis. Interestingly, studies have shown that liver regeneration is dependent on complement. Complement C3 in the proliferative response could be independent of the C3a-C3aR interaction; instead, C3a and C5a appear to act through crosstalk with the local formation of cytokine networks, particularly IL-6 and TNF (177, 355). In addition to complement and CRP, SAA also participates in liver-related diseases. Stereo-seq and scRNA-seq have shown that hepatocytes secrete SAA, facilitating tumor invasion through the recruitment of macrophages that promote M2 polarization (356). Consistent with previous research on pancreatic and colorectal cancers, overexpression of SAA by hepatocytes forms a prometastatic niche in the liver (357). In previous studies, SAA has been reported to exacerbate fatty liver inflammation by promoting intrahepatic platelet aggregation during NAFLD (358). Patients with ethanol-induced liver injury, cirrhosis, and HCC exhibit increased serum levels of SAA and CRP, suggesting an association of these liver-derived innate immune proteins with liver-related diseases (359).

### 4.2 Nervous system diseases

The nervous system stands out as the most intricate and meticulously structured bodily system, governing sensory, behavioral, autonomic, and psychiatric functions across the body. Conversely, the body possesses the ability to influence the host’s brain function and behavior via the “brain-gut axis”. Notably, a mounting body of evidence underscores the liver’s potential to impact the brain, instigating particular behaviors and modulating disease advancement (360). Within the context of inflammatory responses triggered by traumatic brain injury (TBI), Fetuin A—an acute-phase glycoprotein originating from the liver—has been found to hinder microglial necrotic apoptosis, thereby mitigating microglial activation (319). Furthermore, Fetuin A holds potential as a viable biomarker for assessing inflammation in the context of multiple sclerosis (MS) (361, 362). Bile acids, the metabolic byproducts of cholesterol, exhibit associations with immune responses linked to Alzheimer’s disease (AD) and cognitive decline (363). During acute stroke, bile acids exhibit the potential to attenuate glial activation and microglial chemotaxis, suppress MCP-1 and IFN-γ production, and mitigate inflammatory reactions within the central nervous system (CNS) (320). Empirical studies have shown that the administration of tauroursodeoxycholic acid (TUDCA) one hour following ischemia resulting from middle cerebral artery occlusion inhibited perturbations in mitochondrial membranes, extended cell viability, and mitigated both immediate and enduring harm linked to acute stroke (364). In the context of multiple sclerosis (MS), bile acids counteract neurotoxicity and pro-inflammatory polarization in immune cells and glial cells, operating in a dose-dependent manner. Consequently, they lead to diminished TNF-α and IL-1β production (321, 322). The nervous system also stands out as among the most lipid-rich tissues within the human body. Furthermore, PCSK9, functioning as a circulating inhibitor of LDLR, assumes a significant role not only within the CNS but also within the peripheral nervous system (PNS). PCSK9 possesses the capacity to influence immune cell activation within the brain, induce alterations in brain inflammatory responses, and emerge as a novel therapeutic target for addressing brain inflammation (323, 324). Administering PCSK9 inhibitors via intravenous injection can diminish the expression of NF-κB, ameliorate the proliferation and hypertrophy of microglia and astrocytes, and impede neuroinflammation (325). In the EAE model, deficiency of factor FXII was demonstrated to diminish disease-related inflammation concomitantly with heightened Th17-driven production of IL-17A. Namely, factor FXII modifies dendritic cell cytokine profiles to induce effector T cell differentiation, thereby contributing to its role in EAE (365). Simultaneously, thrombin activity within the coagulation system markedly escalates during EAE, demonstrating correlation with the intensity of microglial activation, demyelination, and axonal damage (326). Fibrin deposition is linked to microglial activation in neuroinflammation, and fibrinogen has the capacity to activate microglia, augment their phagocytic capacity, trigger recruitment of peripheral macrophages, and activate Th1 cells (327). These findings imply the feasibility of directing interventions toward the coagulation and fibrinolytic systems for potential anti-inflammatory therapy. The complement system stands as a vital element within the ancestral innate immune response, particularly in degenerative conditions like AD, Huntington’s disease, and Peake’s disease. Several complement components manifest a neuroprotective impact, engaging with complement cell surface receptors, facilitating the elimination of cellular debris and apoptotic cells, and fostering tissue repair via C3a (328).

### 4.3 Cardiovascular diseases

Atherosclerotic plaques are characterized by the accumulation of lipids within macrophages, which culminate in the formation of foam cells. This progression subsequently triggers apoptosis and necrosis of cells within the plaque. Hepatocyte-specific secretion of ANGPTL4 regulates inflammation in atherosclerosis and reduces lipid accumulation in macrophages (329, 330). Hepcidin activates inflammatory response through TLR4 and transcription factor NF-κB. Additionally, it modulates intramacrophage iron efflux by downregulating FPN, thus exerting an impact on atherosclerosis development (331). Patients with atherosclerosis exhibit notably elevated PCSK9 expression in their plasma compared to healthy individuals. Beyond lipid-regulating function, PCSK9 plays a pivotal role in the unfavorable prognosis of this ailment. It not only triggers inflammatory responses, but also fosters thrombotic events and cellular demise. PCSK9 can trigger activation of the TLR4/MyD88/NF-κB signaling cascade, exert influence on the compositional makeup of macrophages, monocytes, and T cells, and augment the release of pro-inflammatory cytokines (IL-1β, IL-6, TNF-α), thereby instigating tissue damage (332). Inhibitors of PCSK9 can regulate inflammatory cell infiltration by regulating macrophage polarization *in vivo* and *in vitro*, resulting in diminished infarct area in cases of acute myocardial infarction (AMI) and enhanced cardiac function (366). Recent investigations have unveiled the influence of PCSK9 on the localized inflammatory response of grafts, underscoring its potential as a therapeutic target to mitigate rejection (76, 138). Coagulation factor XI influences the cleavage of the extracellular matrix bone morphogenetic protein 7 (BMP7) within the heart. Furthermore, it exerts inhibitory effects on the inflammatory response in cases of heart failure, contributing to liver-heart cross-talk (367). Heart failure is closely related to inflammatory states; IL-6 in the inflammatory response is the main trigger for elevated Hepcidin, and iron deficiency due to Hepcidin can also perpetuate and amplify inflammation, including decreased neutrophil activity, defective T cell proliferation, and impaired IL-2 production (333). Moreover, the liver possesses the capacity to influence cardiac repair post-myocardial infarction through the IL-6/STAT3/MR/FGF21 axis (368). FGF21 is an influence not only on adipocytes and renal cells but also plays a role in regulating high blood pressure. Through its promotion of angiotensin-converting enzyme 2 (ACE2) induction, angiotensin II undergoes conversion to angiotensin- (1–7), resulting in the inhibition of hypertension and the reversal of vascular damage. However, inhibiting angiotensin- (1–7) via drug intervention may diminish the safeguarding impact of FGF21 against vascular dysfunction (334). Thrombosis stands out as the most feared complication within cardiovascular disease. The interplay between inflammation and thrombosis orchestrates a sequential activation of platelets and coagulation, culminating in the onset of thrombotic ailments. The crux of immunothrombosis centers on the reciprocal activation of platelets and neutrophils and the liberation of soluble mediators (CCL5, CXCL4, P-selectin, C5a), ultimately increasing vulnerability to ischemic stroke, myocardial infarction, and venous thromboembolism (369).

### 4.4 Autoimmune diseases

FGF21 has demonstrated the capacity to mitigate arthritis severity through the suppression of humoral and cellular immune responses, as well as by dampening the expression of pro-inflammatory cytokines in the cases of rheumatoid arthritis (RA) (335, 336). Abnormal expression of circulating RBP4 has been observed in ankylosing spondylitis (AS), exhibiting a negative correlation with C-reactive protein. This suggests that RBP4 could potentially serve as a biomarker for AS (337). In individuals afflicted with severe psoriasis, Sep levels display significant elevation, which subsequently recedes post-treatment. Given its involvement in modifying anti-inflammatory immune responses, Sep may emerge as a novel indicator for predicting the emergence of inflammatory and metabolic complications in psoriasis (338). Excessive activation of the complement system stands as a paramount contributor to tissue damage in autoimmune disorders. Furthermore, the deficiency of specific complement components can precipitate autoimmune conditions, including systemic lupus erythematosus, vasculitis, Sjögren’s syndrome, and antiphospholipid syndrome (370). Acquired deficiency of C1q is also prevalent among individuals suffering from systemic lupus erythematosus (SLE) (371). Inadequate activation of the complement system culminates in the liberation of pro-inflammatory mediators (C3a and C5a), thereby instigating the recruitment of neutrophils, monocytes, and eosinophils. Additionally, the formation of the MAC triggers inflammation along with cell necrosis or apoptosis, thereby exacerbating tissue damage (339). A Mendelian randomization study showed that the immunomodulatory effects of PCSK9 could have an effect on autoimmune diseases, and that PCSK9 inhibitors significantly reduced the risk of SLE but increased the risk of asthma and Crohn’s disease (372).

### 4.5 Gastrointestinal, hematological, and other types of diseases

ANGPTL4 can modulate intestinal inflammation by impacting macrophage activation and pro-inflammatory cytokine expression (340). Numerous studies have established a connection between selenium (Se) levels and intestinal disease, wherein reduced Se levels were associated with heightened intestinal inflammation and elevated risk of intestinal tumors (341). Overexpression of LECT2 can enhance RIG-I dependent IFN-γ production, bolstering the innate immune response. Targeting LECT2 could potentially hold therapeutic significance in the context of infectious diseases and cancer (373). In cases of sepsis and infectious diseases, elevated Hepcidin levels reflect the activated state of immune cells and correlate with disease severity (342). Over the course of infection, Hepcidin prompts the reduction of extracellular iron levels, which serves as a protective mechanism, inhibiting iron intrusion by pathogens. Conversely, Hepcidin can also encourage the sequestration of iron within macrophages, thereby diminishing the immune response against intracellular infections (144). Furthermore, Hepcidin has notably been linked to inflammatory anemia and inflammatory bowel disease (IBD) (374, 375). The latest evidence suggests that DPP4 inhibitors can reduce the pro-inflammatory and profibrotic responses (including CD8^+^ T cells, macrophages and neutrophils) activated by ANGII in the kidneys, delay kidney damage, and reduce the incidence of proteinuria (376). Paroxysmal nocturnal hemoglobinuria (PNH) is a polyclonal hematologic disorder closely associated with complement reactions. The lack of CD55 and CD59 on the cell surface of the hematopoietic group greatly reduces the complement regulation ability of red blood cells, and after encountering infection, complement is strongly activated, resulting in red blood cells being destroyed by MAC and causing intravascular hemolysis. The prognosis of PNH has been altered after the use of C5 inhibition, and complement inhibitors have also opened up new prospects for the treatment of hematologic diseases (**Table 2**) (343).

## 5 Conclusion and perspectives

As emphasized in this review, the liver, the largest physical organ in the body, serves not only as a metabolic hub but also as a crucial “immune organ.” Within the liver, a diverse array of immune cells and secreted immune molecules converge to establish a complex immune microenvironment, collectively contributing to liver immune regulation. Traditionally, the immunological perspective depicted the liver as a relatively autonomous local immune system influenced by the broader immune network. However, recent advancements in liver immunity research have unveiled the intricate interplay between the liver’s immune function and the body’s overall immune system.

In various pathological and physiological scenarios, the liver orchestrates immune responses within itself and remote organs by releasing hepatokines, innate immune proteins, coagulation factors, and other products into the bloodstream. These immune molecules wield diverse mechanisms for modulating the recognition and elimination of circulating antigens by the innate immune system, thus influencing their capacity to present antigens and initiate adaptive immune responses. Furthermore, immune molecules originating from the liver can directly govern the activation and function of adaptive immune cells. The specific modes of secretion for these signals and their role in sustaining organ function through tightly regulated interactions remain largely unexplored and necessitate further investigation. Simultaneously, intrinsic liver cells and immune cells within the liver capitalize on its distinct circulatory network to detect antigenic constituents coursing through systemic blood circulation and those originating from the gastrointestinal tract, thus engendering immune responses. The liver’s physiological architecture and cellular makeup provide the foundation for temporal and spatial immune tolerance mechanisms. As understanding of the processes of antigen capture, presentation, and recognition within the liver deepens, the clinical application of manipulating liver functions to foster immune tolerance holds increasing promising. Nonetheless, deeper exploration of the intricate interplay among liver immune cells during functional execution and assessment of the strategies of upholding liver immune homeostasis merit additional research.

Evidently, gaining a comprehensive understanding of the intricate, local and distant regulatory mechanisms governing liver immunity could enhance insight into liver function. This knowledge may subsequently uncover novel molecular targets for immune modulation, which could prove to be advantageous for diagnosing and treating immune-related disorders. Moreover, this review lays the groundwork for the exploration and advancement of innovative pharmaceuticals and strategies.

## Author contributions

JW: Funding acquisition, Resources, Writing – original draft, Writing – review & editing. JZh: Writing – review & editing. XZ: Writing – review & editing. YL: Project administration, Validation, Writing – review & editing. JY: Project administration, Validation, Writing – review & editing. ZC: Software, Supervision, Writing – review & editing. YN: Methodology, Supervision, Writing – review & editing. SR: Methodology, Supervision, Writing – review & editing. SW: Supervision, Validation, Writing – review & editing. WY: Supervision, Writing – review & editing. ZL: Validation, Visualization, Writing – review & editing. XL: Project administration, Validation, Writing – review & editing. YH: Project administration, Validation, Writing – review & editing. JZo: Supervision, Validation, Writing – review & editing. CX: Writing – review & editing. JX: Funding acquisition, Resources, Writing – original draft, Writing – review & editing.

## Glossary

**Table:**

LSECs	liver sinusoidal endothelial cells
PD-1	programmed death-1
MHC	major histocompatibility complex
TNF-α	tumor necrosis factor α
LDL-C	low density lipoprotein-cholesterol
TCR	T cell receptor
HTR	heart transplant rejection
GVHD	graft-versus-host
NLRP3	NOD-like receptor thermal protein domain associated protein 3
TLR4	Toll-like receptor 4
TRIF	TIR domain-containing adaptor inducing interferon-β
PPAR-γ	peroxisome proliferator-activated receptor
STAT3	signal transducer and activator of transcription 3
ROR-γt	retinoic acid receptor-related orphan receptor-γt
NF-κB	nuclear factor kappa-B
PI5K	phosphatidylinositol 4-phosphate 5-kinase
ICAM-1	intercellular cell adhesion molecule-1
JNK	c-Jun N-terminal kinase
My88	myeloid differentiation factor 88
TGF-β	transforming growth factor β
IFN-γ	interferon-gamma
